# Co-produced evidence-based recommendations for cascade screening and secondary prevention in the relatives of people diagnosed with non-syndromic thoracic aortic disease

**DOI:** 10.3389/fcvm.2026.1724843

**Published:** 2026-03-31

**Authors:** R. G. Abbasciano, J. C. Dionne, J. Miksza, S. Oczkowski, J. Barwell, N. Shannon, R. Grant, P. Clift, R. Proietti, E. Hope, U. Ahearn, K. Hewitt, L. Ghosh, R. Kaur, M. Lewis, A. Cotton, L. Skinner, H. Saadia, G. McManus, N. Qureshi, H. Aujla, S. Page, R. Sayers, M. Bown, J. Maltby, G. Krasopoulos, D. Cameron, A. Oo, J. Elefteriades, G. Owens, G. J. Murphy

**Affiliations:** 1 Department of Cardiovascular Sciences, University of Leicester, Leicester, United Kingdom; 2 Department of Cardiothoracic Surgery, Imperial College Healthcare NHS Trust, London, United Kingdom; 3 Department of Health Research Methods, Evidence, and Impact, McMaster University, Hamilton, ON, Canada; 4 Leicestershire Clinical Genetics Service, University Hospitals of Leicester NHS Trust, Leicester, United Kingdom; 5 Department of Clinical Genetics, Nottingham University Hospitals, Nottingham, United Kingdom; 6 Department of Cardiology, Queen Elizabeth Hospital Birmingham, Birmingham, United Kingdom; 7 Liverpool Centre for Cardiovascular Science, University of Liverpool and Liverpool Heart and Chest Hospital, Liverpool, United Kingdom; 8 Department of Cardiothoracic Surgery, University Hospital of Southampton, Southampton, United Kingdom; 9 Department of Cardiothoracic Surgery, Liverpool Heart and Chest Hospital, Liverpool, United Kingdom; 10 Department of Cardiothoracic Surgery, University Hospitals Birmingham NHS Trust, Birmingham, United Kingdom; 11 Department of Cardiothoracic Surgery, Barts Heart Centre, St Bartholomew’s Hospital, London, United Kingdom; 12 Department of Cardiothoracic Surgery, John Radcliffe Hospital, Oxford, United Kingdom; 13 Aortic Dissection Awareness UK & Ireland, Woking, United Kingdom; 14 NIHR School of Primary Care Research, University of Nottingham, Nottingham, United Kingdom; 15 School of Psychology and Vision Sciences, University of Leicester, Leicester, United Kingdom; 16 Department of Cardiothoracic Surgery, The Johns Hopkins Hospital, Baltimore, MD, United States; 17 Department of Cardiothoracic Surgery, Yale University School of Medicine, New Haven, CT, United States; 18 Leicester Clinical Trials Unit, University of Leicester, Leicester, United Kingdom

**Keywords:** cascade screening, consensus recommendations, genetic testing, non-syndromic thoracic aortic disease, patient and public involvement

## Abstract

**Objective:**

Over 80% of Thoracic Aortic Disease (TAD) is Non-Syndromic (NS-TAD). However, existing evidence-based guidelines on screening, and secondary prevention are extrapolated from studies in Syndromic-TAD. People with NS-TAD experience unwarranted variation in care. We co-produced evidence-based guidelines for NS-TAD with a view to standardising screening and prevention and identifying gaps in knowledge for future research.

**Methods:**

Using a co-production approach, research questions were defined and ranking using a modified Delphi Process. Evidence based treatment guidelines were developed based on systematic literature reviews, evidence synthesis, and consensus, and used the Grading of Recommendations Assessment, Development and Evaluation (GRADE) approach.

**Results:**

Twelve research questions were selected. Searches screened 7,115 references and identified 121 relevant studies. No studies were identified for 5 questions, so only 7 were subjected to the GRADE synthesis. A strong recommendation was made for routine imaging of all first-degree relatives with NS-TAD. Conditional recommendations with low or very low certainty evidence were made for cascade screening in first- and second-degree relatives, the routine use of combined genetic and imaging for screening, whole exome sequencing over gene panels, and the application of Decision Support Tools to enable shared decision making about cascade screening in families. Research recommendations were made for the comparison of MRI vs. transthoracic echocardiography for cascade screening, and the management of NS-TAD in pregnancy. For secondary prevention, conditional recommendations with low or very low certainty evidence were made for ARBs and Beta Blockers in NS-TAD.

**Conclusions:**

Care of people with NS-TAD is guided by Low certainty evidence.

## 1 Introduction

Thoracic aortic disease (TAD) causes over 2,000 deaths per year in the UK and almost 10,000 in the US (1, 2). The prevalence of the disease is increasing (3). TAD has a long latent phase characterised by asymptomatic aneurysm formation followed by presentation with an acute aortic syndrome, most commonly an acute aortic dissection, which has >70% mortality (4). Early diagnosis, surveillance of aortic size, secondary prevention to slow the rate of aneurysm expansion, and pre-emptive surgery once aortic aneurysms reach a set diameter reduces mortality.

Approximately 20% of TAD is caused by autosomal dominant single gene mutations that have extra-aortic (Syndromic) manifestations enabling early diagnosis (5). Syndromic-TAD is characterised by progressive disease at younger ages that without pre-emptive surgery results in early death from aortic dissection. Cascade screening of the families of people with Syndromic-TAD using imaging and genetic testing is routine, up to 60% of relatives screen positive for the inherited mutation (5), and is associated with significantly increased life expectancy (6).

Almost 80% of TAD is Non-Syndromic (NS-TAD), where disease is restricted to the aorta (7). The aetiology and natural history of NS-TAD is more heterogeneous than Syndromic-TAD, although many phenotypes result in rapid disease progression and early death from aortic dissection. Heritable NS-TAD, characterised by TAD in a first or second degree relative, is caused by inherited single gene mutations. Genes commonly associated with NS-TAD are numerous, and include Vascular Smooth Muscle Cell genes (ACTA2, MYH11, MYLK, PRKG1, FOXE3, MAT2A), Extracellular Matrix genes (LOX, FBN1, MFAP5, THSD4), Transforming Growth Factor-β signalling genes (TGFBR1, TGFBR2, TGFB2, SMAD3, SMAD4, COL3A1), and genes involved in cell development (NOTCH1) (8). Notably, the boundary between syndromic and non-syndromic TAD is not absolute at the genetic level. Several genes implicated in syndromic TAD can also harbour variants that cause isolated, non-syndromic disease, reflecting the pleiotropic nature of pathogenic variants within the same gene (9). In NS-TAD, cascade screening identifies genetic mutations in 15%–30% of first- and second-degree relatives (10). Sporadic NS-TAD, that occurs without a family history, has a heterogeneous aetiology including de-novo gene mutations, polygenic, and environmental causes, can have genetic mutations or aneurysms detectable in up to 25% of first-degree relatives (10). Cascade screening should have important health benefits in NS-TAD and is recommended for people with heritable NS-TAD and disease onset <60 years of age (4, 6, 11). However, these recommendations are not widely implemented. In England, <1% of people diagnosed with NS-TAD subsequently attend a genomic medicine clinic, <50% of people with Hereditary TAD surveyed had undergone genetic testing or cascade family screening, and there is wide regional variation in care (12).

The aim of the current study was to review the evidence for cascade screening and secondary prevention in NS-TAD, develop recommendations to support wider implementation, and identify knowledge gaps for future research.

## 2 Methods

### 2.1 Working group

**Figure 1:**
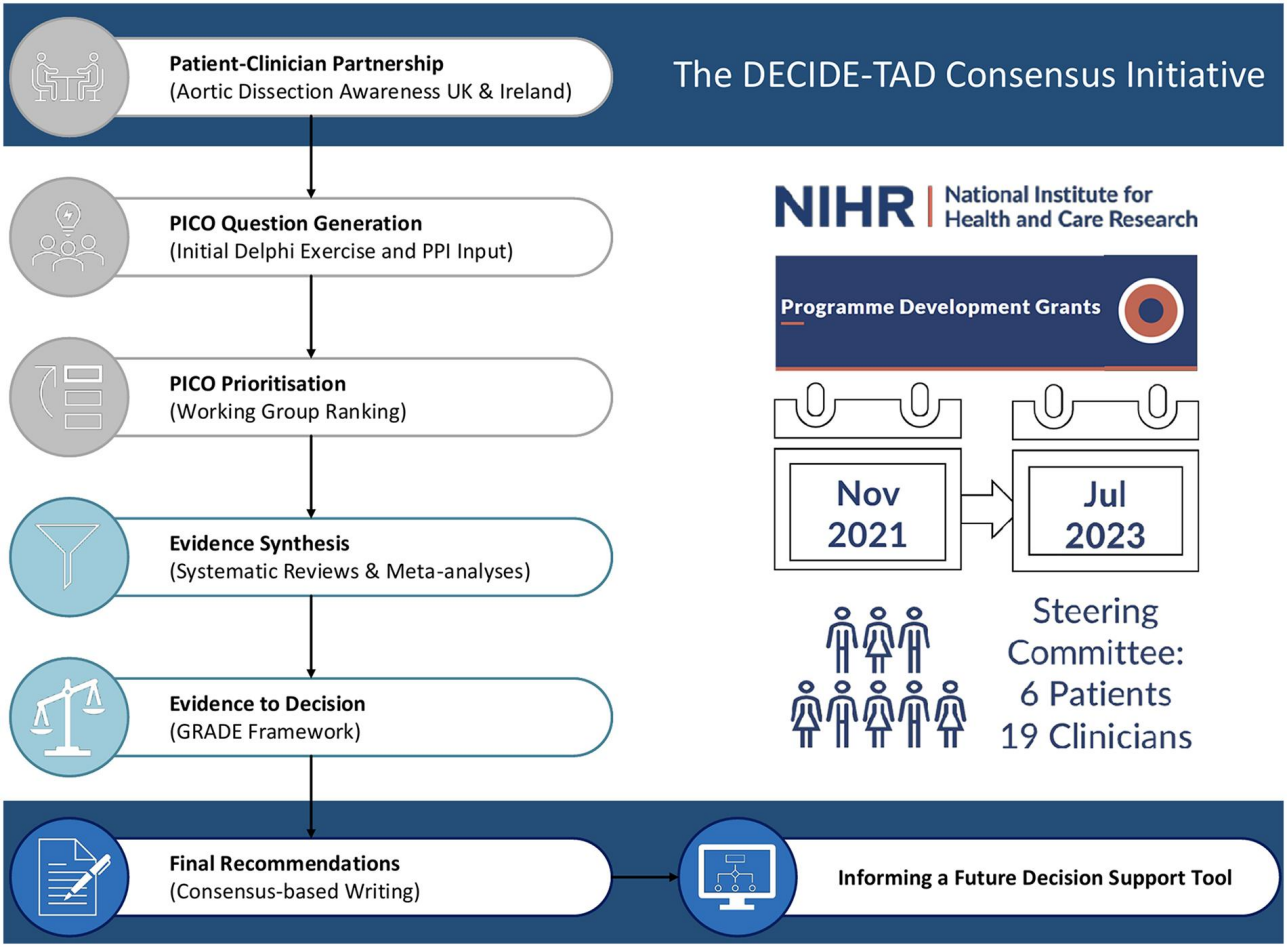
DECIDE TAD guidelines initiative overview. The graphic illustrates the main characteristics and the consecutive stages of the consensus initiative. GRADE, grading of recommendations assessment, development and evaluation; PICO, patient information comparison outcome; PPI, patient and public involvement.

The guidelines were co-produced by six TAD patients and relatives appointed by a patient charity for aortic dissection survivors and their families and carers; Aortic Dissection Awareness in the UK and Ireland (ADA-UK), two clinical geneticists, one primary care physician, two vascular surgeons, six cardiothoracic surgeons, two cardiologists, one psychologist, five specialist aortic nurses and cardiothoracic physician associates. Two expert methodologists in guideline development using Grading of Recommendations, Assessment, Development, and Evaluation (GRADE) (13) assisted the panel in the production of evidence summaries and the process to reach the recommendations from the pooled analyses of the data. Figure 1 illustrates the stages of the initiative. The guideline was produced in accordance with published reporting standard (AGREE Checklist—Supplementary Appendix) (14).

### 2.2 Sponsorship and funding

At each stage of the guideline process, the members of the working group disclosed any possible conflict of interest (COI) (financial, intellectual, or personal). There was no industry involvement in any aspect of the guideline. The project presents independent research funded by the National Institute for Health Research (NIHR203302) and the British Heart Foundation to GJM and HA (CH/12/1/29419) and JM (AA/18/3/34220). The views expressed in this article are those of the author(s) and not necessarily those of the NIHR or the Department of Health and Social Care.

### 2.3 Question development and outcome prioritization

A long-list of 20 PICOs (Population, Intervention, Comparator, Outcome) identified by a Delphi exercise (15) and serial co-production workshops with members of ADA-UK were submitted for ranking to the Working Group, which shortlisted 12 PICOs for evaluation (Supplementary Table S1). Definitions and scope of the recommendations are clarified in Supplementary Table S2.

### 2.4 Search strategy and study inclusion

One author (RGA) developed search strategies for the 12 PICO questions (Supplementary Appendix—Search Strategy for the systematic reviews). Based on the result from exploratory searches, the strategy privileged sensitivity over precision, and general terms for aortopathy were favoured over “non-syndromic thoracic aortic disease” to ensure a higher output of references for screening (foreseeing also a potential need to gather indirect evidence), and implementing a following filtering process that centred the content to be more specific to NS-TAD. Searches from published systematic reviews (SR) were updated to August 2022, and for several PICOs additional literature searches and cross-referencing were performed. The searches were conducted on MEDLINE, EMBASE, and Pubmed and search results were subsequently uploaded into Rayyan for screening (16). Two reviewers (RGA, GJM) screened the search results for relevant studies and systematic review. All references potentially relevant underwent full-text review. Disagreements about relevance and inclusion during this phase were resolved by involving a third member of the WG.

### 2.5 Data extraction (selection and coding)

Details of publications, design and key characteristics of the research work assessed were extracted from eligible studies onto an electronic data collection database. Authors (RGA, SO, JCD, RG) screened the search output independently (Supplementary Table S3) to identify records of potentially eligible studies; the full text of said studies was obtained and assessed. A standardised form was be used for data extraction and included the year and language of publication, country of participants recruited, participant demographic features and nature of treatment/testing analysed, along with indications for treatment, size thresholds, genetic testing, imaging testing, *Z* scores or other BSA indices.

Along with the above list, an additional list of moderators specific to the subdomains of the review was extracted in order to conduct subgroup analyses for the main outcomes (confirmed syndromic condition of the proband, type of underlying syndromic condition, type of participants involved whether First-Degree Relatives or Second-Degree Relatives, type of test performed (imaging/genetic/combined), type of imaging test performed—Computed Tomography (CT)/Magnetic Resonance Imaging (MRI)/Echocardiogram/Multi-modal), type of genetic test performed, setting of the interventions.

The risk ratio (RR) with 95% confidence interval (CI) was calculated for binary outcomes and the mean difference (MD) or the standardised mean difference (SMD) with 95% CI was calculated for continuous outcomes. Summary treatment effect estimates were determined using random effects models. Time-to-event outcomes were summarized through a log-rank approach to obtain hazard ratio. A generic inverse-variance method was used to pool results.

We used the *I*^2^ statistic to measure heterogeneity among the studies in each analysis but acknowledge that there is substantial uncertainty in the value of *I*^2^ when there is only a small number of studies. We considered an *I*^2^ from 0% to 40% as potentially not important, from 30% to 60% as representing moderate heterogeneity, from 50% to 90% as representing substantial heterogeneity, from 75% to 100% as representing considerable heterogeneity.

Risk of bias was assessed using the criteria outlined in the Cochrane Handbook for Systematic Reviews of Interventions (17). Each domain's risk was expressed as high, low or unclear and the risk of bias judgements across different studies were summarised in a “Risk of bias” table (Supplementary Figures S1, S2) and taken into account when considering treatment effects. The Newcastle-Ottawa scale (18) was used for observational studies (Supplementary Table S4). A per-PICO evidence summary is reported in Supplementary Table S5.

### 2.6 Evidence summaries and formulating the recommendations

For each PICO question, the methodology team prepared summaries of evidence that included study design, population, intervention, pooled effect estimates for each outcome, and a rating of the overall quality of evidence. We used the GRADE approach to assess the certainty of evidence for each outcome as “high”, “moderate”, “low”, or “very low”. According to GRADE, we initially assigned a high certainty to evidence from RCTs and a low certainty to evidence from observational data. We then downgraded the certainty by one or two levels if there were serious or very serious concerns about risk of bias (19), inconsistency (20), indirectness (21), imprecision (22), or publication bias. We upgraded the certainty for observational data if there were large effect sizes, dose–response gradients, or plausible confounding. We followed GRADE guidance to consider the relative and absolute effect sizes and to check if the 95% CIs crossed decision-making thresholds. The chairs of the Working Group meeting sessions and methodologists ensured consistency in the judgements of effect sizes across different panel groups for each PICO.

In accordance with the GRADE framework, a “strong” recommendation implies that most patients would want the recommended option, and clinicians would rarely deviate from it. A “weak” or “conditional” recommendation implies that different patients may have different preferences and values, and clinicians would need to consider individual factors when making decisions. Such recommendations may lead to variation in practice.

## 3 Results

We made one strong recommendation and six conditional recommendations. We could not express a recommendation on five PICOs, highlighting the need on further research on these topics. The results are summarised in Tables 1, 2. The evidence summaries for each PICO are provided in the Supplementary Material and are summarised as follows:

**Table 1:** Summary of finding table for the GRADE assessment of the trials evaluating pharmacological interventions for thoracic aortic diseases.

Certainty assessment						No of patients		Effect		Certainty	Importance
No of studies	Risk of bias	Inconsistency	Indirectness	Imprecision	Other considerations	ARB	no ARB	Relative (95% CI)	Absolute (95% CI)		
Mortality—ARB vs. control											
2 RCTs	Not serious	Not serious	Not serious	Very serious	None	0/226 (0.0%)	8/226 (3.5%)	RR 0.11 (0.01–0.88)	32 fewer per 1,000 (from 35 fewer to 4 fewer)	⊕⊕◯◯ Low	CRITICAL
Acute aortic syndromes—ARB vs. control											
5 RCTs	Not serious	Not serious	Not serious	Very serious	None	6/356 (1.7%)	14/339 (4.1%)	RR 0.49 (0.21–1.14)	21 fewer per 1,000 (from 33 fewer to 6 more)	⊕⊕◯◯ Low	CRITICAL
Need for surgery—ARB vs. control											
5 RCTs	Not serious	Not serious	Not serious	Very serious	None	31/358 (8.7%)	31/337 (9.2%)	RR 0.97 (0.61–1.55)	3 fewer per 1,000 (from 36 fewer to 51 more)	⊕⊕◯◯ Low	CRITICAL
Mortality—ARB vs. beta-blocker											
2 RCTs	Not serious	Not serious	Not serious	Very serious	None	3/375 (0.8%)	1/373 (0.3%)	RR 2.33 (0.35–15.58)	4 more per 1,000 (from 2 fewer to 39 more)	⊕⊕◯◯ Low	CRITICAL
Acute aortic syndromes—ARB vs. beta-blocker											
2 RCTs	Not serious	Not serious	Not serious	Very serious	None	3/375 (0.8%)	3/373 (0.8%)	RR 1.00 (0.23–4.35)	0 fewer per 1,000 (from 6 fewer to 27 more)	⊕⊕◯◯ Low	CRITICAL
Need for surgery—ARB vs. beta-blocker											
2 RCTs	Not serious	Not serious	Not serious	Very serious	None	26/375 (6.9%)	19/373 (5.1%)	RR 1.36 (0.77–2.41)	18 more per 1,000 (from 12 fewer to 72 more)	⊕⊕◯◯ Low	CRITICAL
Size change—ARB vs. control											
4 RCTs	Not serious	Not serious	Not serious	Serious	None	326	300	-	MD 0.07 lower (0.11 lower to 0.01 lower)	⊕⊕⊕◯ Moderate	IMPORTANT
Size change—ARB vs. beta-blocker											
3 RCTs	Not serious	Serious	Not serious	Serious	None	364	358	-	MD 0.02 higher (0.05 lower to 0.1 higher)	⊕⊕◯◯ Low	IMPORTANT
Mortality—beta blocker vs. control											
2 RCTs	Not serious	Not serious	Not serious	Very serious	None	1/57 (1.8%)	5/66 (7.6%)	RR 0.32 (0.05–1.87)	52 fewer per 1,000 (from 72 fewer to 66 more)	⊕⊕◯◯ Low	CRITICAL
Acute aortic syndromes—beta blocker vs. control											
2 RCTs	Not serious	Not serious	Not serious	Very serious	None	3/57 (5.3%)	6/63 (9.5%)	RR 0.56 (0.15–2.13)	42 fewer per 1,000 (from 81 fewer to 108 more)	⊕⊕◯◯ Low	CRITICAL
Disease progression beta blocker vs. control											
2 RCTs	Not serious	Not serious	Not serious	Very serious	None	6/57 (10.5%)	15/66 (22.7%)	RR 0.45 (0.20–1.02)	125 fewer per 1,000 (from 182 fewer to 5 more)	⊕⊕◯◯ Low	CRITICAL

The table reports the summary of findings for PICOs related to pharmacological interventions to treat thoracic aortic disease. With the exception of one outcome (size change in ARB vs. control, moderate level of certainty) the level of certainty was low. ARB, angiotensin receptor blocker; CI, confidence interval; MD, mean difference; RR, relative risk.

**Table 2:** Screening, diagnostic and therapeutic strategies in relatives of patients with aortic diseases.

Question	Recommendation	Justification
Screening		
Should *non-selective* imaging screening tests for *NS-TAD be restricted to* first-degree relatives (FDRs) *only* vs. *FDR and* second-degree relatives (SDRs)?	We suggest the use of imaging screening in FDRs and SDRs (conditional recommendation, very low certainty of evidence).	Patients and relatives prefer to know their risk status and feel reassured by negative tests. The panel recommends screening for first- and second-degree relatives but acknowledges the limitations of the evidence and the uncertainties of clinical outcomes.
*Non-selective genetic testing vs. selective genetic testing of probands with NS-TAD in addition to unselected imaging?*	We recommend the non-selective use of genetic testing in probands with NS-TAD (conditional recommendation, very low certainty of evidence).	Clinicians in the panel described how the major limitation in the available evidence is the lack of follow up data. Patient members of the panel felt that the presence of an additional secondary test is a valuable form of reassurance, despite the risk of finding variants of unclear significance.
*Gene panels (any) vs. whole exome sequencing for genetic screening in NS-TAD?*	We suggest using whole exome sequencing over gene panels in screening family members of individuals with non-syndrome thoracic aortic disease (conditional recommendation, low certainty).	Whole exome sequencing reduces the selection bias of gene panels and it can find more cases of non-syndromic TAD, but it can also lead to overdiagnosis and false reassurance. The panel recognises how patients’ preferences and values should be taken in consideration.
*Selective vs. non-selective imaging in first-degree relatives of people with NS-TAD?*	We recommend extensive adoption of imaging screening in first degree relatives of patients with non-syndromic thoracic aortic disease (strong recommendation, low certainty of evidence).	The high diagnosis rate in first degree relatives—with over 1 in 4 relatives having an aortic aneurysm provides an opportunity to avoid a catastrophic event and carries minimal patient harm.
Surveillance and diagnosis modalities		
*Decision support tools (DSTs) vs. usual care for shared decision making in family screening for NS-TADs?*	We suggest using DSTs for survivors of acute aortic syndromes and their families to help guide screening and treatment decisions (conditional recommendation, low certainty evidence).	DSTs are designed to help patients make informed choices that align with their goals and improve their care outcomes. However, most of the existing decision aids assessed were focused on patients with abdominal aortic aneurysms. The quality and accessibility of the tools may also affect their impact.
*Should echocardiography be used in preference to MRI for screening people at risk for NS-TAD?*	We could not make a recommendation for the use of echo or MRI for screening aortopathy given the lack of evidence comparing the two modalities (no recommendation, very low certainty of evidence). The panel highlighted that patients with type B dissection would be missed on screening with echo compared to MRI screening.	The panel highlighted during review of the evidence that there are potential inequities that exist between these screening modalities. Data is lacking on cost of resources required and cost effectiveness of using one modality compared to another.
*Pre-test genetic counselling vs. standard care in families of people with acute aortic syndromes?*	No studies were identified to inform this question examining these interventions.	The impact of pre-test genetic counselling for patients and families affected by acute aortic syndromes is a research priority.
*Routine aortic angio-MRI vs. no routine aortic angio-MRI in women at risk of NS-TAD who are planning a pregnancy?*	We could not formulate a recommendation on the specifics of prevention during pregnancy in women with a family history of aortic disease, due to the lack of studies. This topic constitutes a research priority, both for the burden of the disease and for the extent of the evidence gap.	In the absence of research conducted in patients with non-syndromic aortopathy, guidance for the management of pregnancy in aortopathic patients with syndromic conditions (in particular Marfan's syndrome) (10, 11) can be used as an indirect source of recommendations.
Pharmacological management		
*Angiotensin receptor blockers (ARB) vs. no angiotensin receptor blockers for patients at risk of NS-TAD?*	We suggest using an ARB in patients with Marfan's syndrome, whether or not they are already on a Beta-blocker (conditional recommendation, low certainty evidence).	Patients with aortopathy, especially Marfan syndrome, may benefit from ARBs to slow down aneurysm growth and reduce mortality, but the evidence is weak. ARBs may also cause side effects, and while lower doses may help, but their effectiveness is unknown.
*Beta-blockers vs. no beta-blockers for patients at risk of NS-TAD?*	We suggest using Beta-blockers in patients at risk for aortopathy, specifically those with Marfan's syndrome (conditional recommendation, low certainty evidence).	Beta-blockers may slow aortic growth and have few side effects. However, this recommendation is weak, because the evidence is limited, mostly from Marfan syndrome patients.
*Pharmacologic cardiovascular risk management (anti-platelet agents, lipid lowering, glycaemic control) compared with any control or placebo, in patients with NS-TAD*	We cannot make a recommendation for the use of antiplatelet medication in this patient population. We suggest screening for diabetes and having glycemic control in those with aortic diseases to prevent complications as per general cardiovascular risk prevention (Conditional recommendation, very low certainty of evidence). We cannot make a recommendation at this time for the use of lipid control in this population (Very low certainty of evidence).	There is no evidence for the use of antiplatelet therapy in this patient population. Use of antiplatelet therapy should be used based on other cardiovascular risk factors and recommendations in prior guidelines for the use of antiplatelet therapy in patients with known atherosclerotic disease. The unexpected finding that diabetes may actually have a protective effect against aortic dissection has attracted considerable attention and controversy among researchers, with inconsistent conclusions, but currently the impact of antidiabetic treatments on TAD development and progression remains uncertain. Lipid control could be considered in this patient population based on individual factors, but data is lacking on the effect of lipid control in this population on primary aortic dissection prevention.
*Angiotensin Converting Enzyme inhibitors (ACEi) vs. no Angiotensin Converting Enzyme inhibitors for patients at risk of NS-TAD?*	We made no recommendation on the use of ACEi in thoracic aortopathy due to the lack of evidence.	The WG suggested to consider the use of ARB instead given there is more evidence for this, as described above, at least in patients with Marfan's.

The table lists the PICOs discussed in the evidence synthesis exercise and the relative recommendations produced by the panel, along with a summary of the justification for each statement. The extended discussion according to the GUIDE evidence to decision framework is available in the Digital Supplementary Appendix. ACEi, angiotensin converting enzyme inhibitors; ARB, angiotensin receptor blocker.

### 3.1 Selective vs. non-selective imaging in first-degree relatives of people with NS-TAD?

#### 3.1.1 Recommendation

We strongly recommend routine imaging screening in all first-degree relatives of patients with NS-TAD (strong recommendation, low certainty of evidence).

#### 3.1.2 Evidence summary

We identified 5 observational studies to assist us in replying to this question (7, 23–26). Imaging of unselected first-degree relatives of patients affected by NS-TAD may result in a diagnosis rate of 26% (95% CI 0.16–0.4). Patients’ depression symptoms (assessed with: PHQ-9) and anxiety symptoms (assessed with GAD-7) were unchanged before and after screening (3 months follow up) according to a single observational study (7). The overall level of certainty for the outcomes assessed was low, due to the methodological limitations of the studies included (observational studies) and limited sample size. Statistical inconsistency could be suspected based on the significant I-squared value (56%), although the point estimates were clinically similar across studies.

#### 3.1.3 Justification

Although the certainty of direct evidence linking screening to reduced mortality is low, the panel issued a strong recommendation based on three key factors: high diagnostic yield, avoidance of catastrophic harm, patient values and preferences. First degree relatives (FDR) of patients who present with NS-TAD are at increased risk of having TAD (10). Existing guidelines recommend restricting imaging screening to FDR of those with risk factors for Heritable disease (4). However in a small unselected cohort of probands with Hereditary and Sporadic NS-TAD one study, the diagnosis rate for new disease with imaging in FDR was 25% (7). In a cohort of patients with Sporadic NS-TAD new disease was identified in 1 in 6 FDR (26). By comparison, incidental rates of diagnosis of TAD (defined as thoracic aorta diameter ≥4.0 cm) on CT scans performed at a single hospital on 5,662 patients (27) was 2.1% (rates increased with age, with 5.7% of males over 84 years old having aortic root dilation of ≥4.5 cm). Given the low risks of imaging and likely benefits of early diagnosis of NS-TAD, patient members of the working group expressed strong views that routine imaging of all FDR of probands with NS-TAD should yield a net benefit.

#### 3.1.4 Implementation issues

There is no direct evidence that non-selective screening leads to earlier treatment or better clinical outcomes. Unselected screening of relatives also requires more resources than selective screening, both in the initial tests as well as follow-up monitoring and secondary prevention and psychological support. The cost-effectiveness of unselected screening is uncertain. There may also be regional variation in the availability of screening (12, 15).

For the purpose of this recommendation, “routine screening” is defined as a TTE visualizing the aortic root and ascending aorta, with escalation to cross-sectional imaging in case of inadequate windows or suspicion of disease involving the distal aorta.

The panel acknowledged the potential for harms, primarily related to resource use and false positives. In a recent observational study, 68% of familial relatives required some form of subsequent management or surveillance, increasing the burden on healthcare systems (7). While false positives (diagnosed by Echo but refuted by MRI) occurred in a minority of cases, they can lead to temporary anxiety and costs associated with confirmatory imaging. However, the evidence synthesis found no significant increase in depression (PHQ-9) or anxiety (GAD-7) scores among screened relatives.

### 3.2 Should non-selective imaging screening tests for NS-TAD be restricted to first-degree relatives (FDRs) only vs. FDR and second-degree relatives (SDRs)?

#### 3.2.1 Recommendation

We suggest the use of routine imaging screening for first-degree relatives and second-degree relatives (conditional recommendation, very low certainty of evidence).

#### 3.2.2 Evidence summary

The certainty of the evidence is reduced by the small sample size and methodological limitations of the included observational studies (23–25, 28) (Supplementary Figures S3, S4).

In one small study, the average diagnosis rate with imaging tests among FDRs was 16% (95% CI 7%–33%) compared to 18% (95% CI 5%–46%) when screening both FDRs and SDRs (7). There were no pre-/-post differences in the effect of screening on quality of life or anxiety and depression scores between FDRs and SDRs (although the study used a combined approach to screening, with genetic and imaging testing). There was no data to assess whether screening reduced the incidence of aortic dissection in SDR.

#### 3.2.3 Justification

Screening in SDR is likely to yield substantial numbers of new cases of TAD with no evidence of increased psychological stress. Patient members of the panel described how not knowing whether you have TAD has probably a worse impact on quality of life than knowing, and judged the reassurance offered even from a negative result as a desirable outcome.

#### 3.2.4 Implementation considerations

The availability of different imaging modalities might create barriers to access or variations in uptake of screening, but the available evidence did not allow us to address these aspects of the question. Moreover, there are no formal cost and cost-effectiveness studies to inform practice.

### 3.3 Should echocardiography be used in preference to MRI for screening people at risk for NS-TAD?

#### 3.3.1 Recommendation

We could not make a recommendation due to the lack of evidence comparing the two modalities (no recommendation, very low certainty of evidence).

#### 3.3.2 Evidence summary

One study (7) compared screening for NS-TAD with echocardiograms vs. MRI in 54 relatives of 16 probands with heritable or sporadic NS-TAD. There was no difference between the two modalities RR 0.98 (95% CI 0.42–2.26). Given few events and wide confidence intervals, the overall certainty of evidence was low.

#### 3.3.3 Justification

NS-TAD affecting the distal ascending aorta, aortic arch, and descending aorta may be missed with echography alone, where aortic root pathology will be identified. MRI screening provides accurate information on the entirety of the thoracic aorta, as well as potentially meaningful additional information on aortic distensibility and tortuosity, which transthoracic echocardiography (TTE) cannot offer.

#### 3.3.4 Implementation

Both echocardiography and MRI are currently used for family screening in NS-TAD. The panel identified potential inequities that exist between these screening modalities. Echocardiography is more likely to be available than MRI in low resource healthcare systems. Data is lacking on the differences in cost effectiveness of using one modality compared to another. More research is needed, however if MRI screening is indicated as the primary screening modality this would require service reconfiguration and investment to ensure equity of access. The most recent AHA Guidelines (4) recommend performing either MRI or CT in patients with an incomplete visualization of the aorta obtained from TTE.

### 3.4 Routine aortic angio-MRI vs. no routine aortic angio-MRI in women at risk of NS-TAD who are planning a pregnancy?

#### 3.4.1 Recommendation

We could not formulate a recommendation for this question due to a lack of evidence.

#### 3.4.2 Evidence summary

The risk of aortic dissection is four times higher; incidence rate ratio 4.0 (95% confidence interval, 2.0–8.2) during pregnancy than in the first year after pregnancy (29). In the IRAD registry, 29 cases occurred in pregnant women, 0.3% of the total, and 1% of aortic dissections in women. Most women (69%) had an underlying syndromic aortopathy or a positive family history (15%), and half were not aware of their condition until after the aortic dissection (30). The onset time was during pregnancy for 15 women (4 in the first trimester and 11 in the third trimester) and after delivery for 12 women (mean of 12.5 ± 14 days post-partum).

In the absence of research conducted in women with NS-TAD who are planning a pregnancy, guidance for the management of pregnancy in Syndromic-TAD (31, 32) can be used as an indirect source of recommendations. Women with Syndromic-TAD should undergo maternity care by multidisciplinary teams in specialist centres. Serial imaging with transthoracic echocardiography is recommended each trimester and postpartum; if more extensive aortic visualization is necessary, MRI without gadolinium is the preferred modality over CT (4).

#### 3.4.3 Implementation

Until further research becomes available, clinicians should carefully examine family history and clinical signs of TAD and choose an individualised approach to select timing and imaging modality in women at risk of NS-TAD who are pregnant or are planning pregnancy (33).

#### 3.4.4 Research priorities

This topic constitutes a research priority, both for the burden of the disease and for the lack of direct evidence to guide clinical decision making.

### 3.5 Non-selective genetic testing vs. selective genetic testing of probands with NS-TAD in addition to unselected imaging?

#### 3.5.1 Recommendation

We recommend the non-selective use of genetic testing in probands with NS-TAD (conditional recommendation, very low certainty of evidence).

#### 3.5.2 Evidence summary

No randomised controlled trial was identified. Among 40 observational studies (7, 23–26, 28, 34–67), combining genetic testing with imaging testing may not increase the diagnostic yield (OR 0.93, 95% CI 0.77–1.12, very low certainty of evidence for inconsistency across studies, statistical heterogeneity and low number of events). Uptake of the testing was probably higher when genetic and imaging testing were offered jointly (OR 1.22, 95% CI 1.00–1.50, very low certainty of evidence for inconsistency across studies, statistical heterogeneity and low number of events) (Supplementary Figures S3, S4).

Thijssen and colleagues (68) recently performed a health-related quality of life (HRQOL) assessment in 147 patients with TAD, 114 screening participants and 66 partners. The study found that probands with TAD had lower HRQOL, however, families being screened were not as affected.

#### 3.5.3 Justification

Existing guidelines recommend selective genetic testing in probands with risk factors for Heritable disease (4). Clinicians in the panel described how the major limitation in the available evidence is the lack of follow up data on the effects of non-selective genetic testing on long term clinical outcomes that could justify the implementation of combined imaging and genetic screening programmes. Nonetheless, clinicians agreed on the benefit deriving from added genetic information in choosing management options for familiar forms of the disease. Patient members of the panel felt that unselected genetic testing in addition to imaging was a valuable form of reassurance and therefore carries significant desirable effects, despite the risk of finding variants of unclear significance during a genetic test.

#### 3.5.4 Implementation considerations

Costs deriving from the implementation of an unselected combined approach to screening in TAD might be significant, but no direct evidence is available for a cost-effectiveness analysis.

### 3.6 Pre-test genetic counselling vs. standard care in families of people with acute aortic syndromes?

#### 3.6.1 Recommendation

We could not make a recommendation for this question. Regardless of the timing when offered, the panel recognises the importance of counselling for families with potentially inheritable forms of aortic disease.

#### 3.6.2 Evidence summary

Modalities, frequency and timing of genetic counselling offer differ widely across the UK according to patients' experience (15), and pre-test counselling is not routine. A small study (*n* = 10) reported the effects of disclosure of clinically actionable genetic variants to thoracic aortic dissection biobank participants who underwent whole exome sequencing, with clinical genetic counselling and confirmatory genetic testing in a CLIA laboratory (69). The participants reported low levels of psychological distress following genetic counselling (the average FACToR score was 16.0 ± 4.2, out of 100). The subscale scores were also low for negative feelings (3.7 ± 3.4; range 0–12, out of 12), uncertainty (2.0 ± 1.7; range: 0–5, out of 8), and privacy concerns (1.7 ± 2.0, range 0–5, out of 8). Positive feelings were around the midpoint, indicating a moderate level of positive emotional responses to the genetic test result (8.7 ± 3.8; range 0–12, out of 16). The average decisional regret scores, 11.5 ± 11.6 (range 0–25, out of 100) indicate low levels of regret about deciding to learn their genetic test results.

#### 3.6.3 Implementation considerations

In one study the total expense per person for genetic testing, verification, and mailing results was US$400, whereas the total expense per person who got genetic counselling and confirmation from a certified lab was US$605 (69).

#### 3.6.4 Research

The impact of pre-test genetic counselling for patients and families affected by NS-TAD should be considered a research priority.

### 3.7 Gene panels (any) vs. whole exome sequencing for genetic screening in NS-TAD?

#### 3.7.1 Recommendation

We suggest whole exome sequencing over gene panels in screening family members of individuals with NS-TAD (conditional recommendation, low certainty).

#### 3.7.2 Evidence summary

Six observational studies adopting testing with a gene panel in NS-TAD reported an average test positive rate of 21% (95% CI 16%–26%) in a total of 390 participants (Supplementary Figures S5, S6). In eight observational studies whole exome sequencing resulted in an average test positive rate of 30% (95% CI 23%–39%) in 278 people with NS-TAD.

#### 3.7.3 Justification

Whole exome sequencing may yield higher detection rates, a desirable outcome for some patients. Conversely, a higher diagnosis rate obtained through whole exome sequencing in the absence of a clinically actionable result might not necessarily correspond to a desirable effect. The risks of false reassurance from negative tests may be higher with whole exome sequencing (perceived as a more definitive test). On balance, underdiagnosis might pose a more serious and catastrophic risk, although there no direct evidence to support this. Whole exome sequencing can also facilitate research, or diagnosis at a later time without the need for retesting.

#### 3.7.4 Implementation considerations

Gene panels are easier to implement and require and less resources. Although whole exome sequencing is becoming progressively less expensive, there is still a significant increase in the amount of time required to review its results, in particular in terms of the human resources and time required.

Lastly, whole exome sequencing may help address the inequity in recruitment in the exisiting studies of causal genes (and the resulting gene panels). Ranking of items listed on contemporary gene panels is based on observational studies and case reports, performed typically in countries with a stronger research activity, and potentially missing variants more common in cohorts that are less frequently taking part in scientific studies. Whole exome sequencing could be more appropriately and effectively implemented in diverse groups within a multicultural setting.

A higher diagnosis rate must be balanced against the higher risk of identifying VUS or incidental findings. WES is superior only when supported by an infrastructure that can reliably avoid false reassurance or unnecessary anxiety.

### 3.8 Decision support tools (DSTs) vs. usual care for shared decision making in family screening for NS-TADs?

#### 3.8.1 Recommendation

We suggest using DSTs for people at risk of NS-TAD who are considering cascade screening (conditional recommendation, low certainty evidence).

#### 3.8.2 Evidence summary

The available evidence is indirect. Studies evaluating Decision Aids for people with AAA making treatment choices (70–72) showed that they did not have a significant effect on patients' satisfaction (SMD −0.06, 95% CI −0.25 to 0.13, 3 studies, low certainty evidence), decisional conflict (MD −2, 95% CI −7.38 to 3.38, 2 studies, low certainty evidence), anxiety (SMD −0.07, 95% CI −0.33 to 0.2, 2 studies, low certainty evidence), or quality of life (MD −1, 95% CI −4.06 to 2.06, 1 study, low certainty evidence). However, evidence suggests that DSTs may improve patients' understanding of their treatment choices (SMD −0.27, 95% CI −0.54 to 0.01, 2 studies, low certainty evidence).

#### 3.8.3 Justification

While the anatomical location differs, the decisional process for patients with AAA and NS-TAD is clinically similar. Both groups face in fact the choice between the immediate risks of elective surgery and the future uncertain risk of adverse events. Extrapolation may however be limited by demographics. The AAA population is typically older and male, whereas NS-TAD affects a younger, more gender-balanced cohort where issues of career, insurance, and child-bearing are more prominent.

Decisions about cascade screening in people with NS-TAD are influenced by personal values and preferences, as well as the risks and benefits of different interventions. Decision support tools are designed to help patients make informed choices that align with their goals and improve their care outcomes. There is no direct evidence on how effective decision support tools are for patients with NS-TAD.

Most patients in the working group considered decision support tools as helpful. DSTs may also increase equity by providing more standardized information sharing and decision-making for patients. This depends on whether or not the tools are provided equitably and in a consistent way.

#### 3.8.4 Implementation issues

There are moderate increases in costs involved in designing, implementing and updating these tools, which may require more resources than simply providing usual care. DSTs result in longer consultation times (72), although this was considered a positive outcome from the patient's perspective, as they had more time to review and discuss their options. The cost-effectiveness of the decision support tools will depend on whether they increase or decrease the overall costs of care, which are uncertain. Patients on the panel were more likely accept the use of decision support tools, if they supplemented and reinforced current care, but not if they replaced current processes (e.g., meeting with the clinical team).

#### 3.8.5 Research priorities

Research is needed to develop and evaluate decision support tools that are tailored to people at risk of NS-TAD making decisions about cascade screening.

### 3.9 Beta-blockers vs. no beta-blockers for patients at risk of NS-TAD?

#### 3.9.1 Recommendation

We suggest using Beta-blockers in patients at risk of NS-TAD (conditional recommendation, low certainty evidence).

Conditions for the recommendation are first, all of the available evidence is indirect, as it includes only studies involving patients with Marfan syndrome, so the effects of Beta-blockers on NS-TAD is unclear. Second, the recommendation should be considered in the context of other treatment options that are available for NS-TAD, such as angiotensin receptor blockers (ARBs), which have also shown promising results in reducing aortic root growth and may have additive effects with Beta-blockers.

The extrapolation of pharmacological efficacy from syndromic forms to NS-TAD is supported by biological plausibility, as regardless of the specific genetic driver, reducing parietal tension via blood pressure control (Beta-blockers/ARBs) theoretically mitigates wall stress and expansion. Extrapolation is however less confident in specific subtypes, such as *ACTA2* or *MYH11* mutations, where the disease mechanism involves smooth muscle contraction rather than extracellular matrix signalling, and where dissection may occur at smaller diameters.

#### 3.9.2 Evidence summary

Three studies (73–75) provided the data to formulate this recommendation (Supplementary Figures S11–S13). The meta-analysis by Gersony et al. (76) was also assessed. All the studies were conducted among participants with Marfan syndrome. Quantitative synthesis demonstrated that Beta-blockers did not reduce mortality (RR 0.32, 95% CI 0.05–1.87, 2 studies, low certainty of evidence), incidence of acute aortic syndromes (RR 0.56, 95% CI 0.15–2.13, 2 studies, low certainty of evidence), and had an unclear effect on death or cardiovascular events (RR 1.42, 95% CI 0.91–2.22, 5 studies, very low certainty of evidence). Beta-blockers were likely to reduce disease progression, measured as a dichotomous event (RR 0.45, 95% CI 0.20–1.02, 2 studies, low certainty of evidence).

Ong et al. (74) reported that 12% of the participants (3 out of 25) felt tired. Shores et al. (75) found that 33% of the participants (10 out of 30) who received propranolol, a non-selective Beta-blocker, had adverse effects such as heart block, lethargy, depression, insomnia, dream disturbance, bronchospasm, and increased sensitivity to alcohol.

#### 3.9.3 Justification

Beta-blockers appear to reduce expansion of aortic root aneurysms in Marfan Syndrome, but evidence has not demonstrated a reduction in rates of dissection or the need for surgical repair. The adverse effects of beta-blockers were minimal and mostly tolerable. Modern cardio-selective Beta-blockers, which target only specific receptors in the heart, have fewer side effects than the non-selective Beta-blockers evaluated in these studies. The applicability to NS-TAD is uncertain, as the relationship between aortic diameter and dissection is more heterogeneous than for Marfan Syndrome. However, on balance, given the similar disease mechanisms underlying Syndromic and many types of NS-TAD there are likely to be benefits (4). There are also well demonstrated benefits of Beta-blockers in people with risk factors for disease progression such as hypertension. Given the uncertainty, patients may have different preferences and values that should be considered in the shared decision-making process with their clinicians when considering Beta-blocker therapy.

#### 3.9.4 Implementation considerations

A significant barrier to implementation is patients' willingness to take life-long preventive medications, particularly given the limitations of the evidence for their use in NS-TAD. A second barrier is that cumulative cost of antihypertensive drugs over a life-time can be significant whereas the it is unclear whether these are offset by reducing the occurrence of adverse events that require surgery, but we are uncertain about the overall balance of costs and savings.

### 3.10 Angiotensin receptor blockers (ARB) vs. no angiotensin receptor blockers for patients at risk of NS-TAD?

#### 3.10.1 Recommendation

We suggest using an ARB in patients with NS-TAD, whether or not they are already on a Beta-blocker (conditional recommendation, low certainty evidence).

Conditions for this recommendation includes that the evidence is indirect, as all the trials enrolled patients with Marfan syndrome. The choice of whether to start an ARB medication (and the specific agent) for these patients can at present be guided by the anticipated mechanism of effect (e.g., genetics/pathology vs. shear stress etc.) until further evidence is available.

#### 3.10.2 Evidence summary

A total of 8 randomised controlled trials were retrieved from literature searches (77–84), along with a recent individual patient-data meta-analysis (85) (Supplementary Figures S7–S10). All the trials were conducted in patients affected by Marfan syndrome. When ARB were compared with placebo, the majority or the trial participants (75% in both arms) were on a concomitant treatment with Beta-blockers. ARB reduced the risk of mortality compared with placebo (RR 0.11, 95% CI 0.01–0.88, 2 studies, low certainty of evidence); the effect of ARB on mortality was unclear when compared to Beta-blocker treatment (RR 2.23, 95% CI 0.35–15.58, 2 studies, low certainty of evidence). ARBs had an unclear effect on the incidence of acute aortic syndromes, vs. control (RR 0.49, 95% CI 0.21–1.14, 5 studies, low certainty of evidence) and Beta-blocker treatment (RR 1.00, 95% CI 0.23–4.35, 2 studies, low certainty of evidence), and on the need for surgery, vs. control (RR 0.97, 95% CI 0.61–1.55, 5 studies, low certainty of evidence) and Beta-blocker treatment (RR 1.36, 95% CI 0.77–2.41, 2 studies, low certainty of evidence). Increase in aneurysmal size (measured as body surface area-adjusted aortic root dimension *Z* score) was marginally lower with ARB treatment vs. control (MD −0.07, 95% CI −0.11 to 0.01, 4 studies, moderate certainty of evidence), and unchanged in ARB vs. Beta-blocker treatment (MD −0.02, 95% CI −0.05 to 0.1, 3 studies, moderate certainty of evidence).

Adverse event rates differed across trials, but with similar incidence between intervention and control group.

#### 3.10.3 Justification

Patients with NS-TAD highly value interventions that may lower their risk of death or need for surgery. Data suggesting a reduction in mortality and a minor decrease in aneurysm growth in Marfan Syndrome, without significant adverse effects provides indirect evidence of effectiveness in NS-TAD.

#### 3.10.4 Implementation considerations

Compared to Beta-blockers, ARBs are more expensive and require regular monitoring of renal function, resulting in slightly higher resource usage. However, the difference in cost is small. Indirect evidence of cost-effectiveness of anti-hypertensives to treat hypertension suggest that ARBs may be more cost effective than Beta-blockers, and that either is more cost-effective than no treatment (86). However, in patients without pre-existing hypertension, these comparisons likely no longer apply.

There may be variability regarding patients' willingness to take medications for future event prevention, depending on their understanding and appreciation of prognosis, engagement in their own health, experience with illness, and stage of life. For instance, younger patients may value prevention and side effects differently than older patients. These differences may be similar or even greater for Beta-blockers.

### 3.11 Angiotensin converting enzyme inhibitors (ACEi) vs. no angiotensin converting enzyme inhibitors for patients at risk of NS-TAD?

#### 3.11.1 Recommendation

We could not formulate a recommendation on the use of ACEi in NS-TAD.

#### 3.11.2 Evidence summary

No studies evaluating ACEi in TAD were identified.

#### 3.11.3 Justification

The lack of direct evidence prevented us from producing a recommendation for this question.

#### 3.11.4 Research priorities

Randomised controlled trials of ACEi in NS-TAD are a current research gap.

### 3.12 Pharmacologic cardiovascular risk management (anti-platelet agents, lipid lowering, glycaemic control) compared with any control or placebo, in patients with NS-TAD

#### 3.12.1 Should antiplatelet therapy vs. placebo be used for aortic syndromes?

##### 3.12.1.1 Recommendation

We cannot make a recommendation for the use of antiplatelet medication in this patient population.

##### 3.12.1.2 Evidence summary

Although there is an extensive amount of literature in regard to antiplatelet agents for general cardiovascular risk factor risk reduction, there is no direct evidence that considers this strategy in patients NS-TAD. Indirect evidence from studies in small AAA do not demonstrate reduction in aneurysm growth with either aspirin (87) or Ticagrelor (88).

##### 3.12.1.3 Justification

Use of antiplatelet therapy should be used based on other cardiovascular risk factors and recommendations for the use of antiplatelet therapy in patients with known atherosclerotic disease. Recent guidelines support the use of low-dose aspirin in patients with thoracic aortic aneurysms in presence of documented atherosclerosis or penetrating aortic ulcers (4).

##### 3.12.1.4 Research priorities

Randomised controlled trials of anti-platelets in NS-TAD are needed.

#### 3.12.2 Should glycaemic control vs. placebo be used for aortic syndromes?

##### 3.12.2.1 Recommendation

Glycaemic control should be pursued as per general cardiovascular risk prevention.

##### 3.12.2.2 Evidence summary

Cardiovascular diseases and atherosclerosis are more likely to occur in people with diabetes, as high blood glycaemic levels can damage the blood vessels and increase the risk of complications. Moreover, hyperglycaemia can also worsen the outcomes of microvascular problems, such as kidney disease and eye damage, in diabetic patients. Therefore, one might expect that diabetes would also increase the risk of aortic dissection. However, several recent studies have challenged this assumption and suggested that diabetes may actually have a protective effect against aortic dissection (89–91).

##### 3.12.2.3 Justification

The associations between diabetes and TAD progression are inconsistent. The impact of antidiabetic treatments on NS-TAD development are unknown (92).

##### 3.12.2.4 Research priorities

Several observational and experimental studies have pointed to a potential protective effect of metformin in AAA (93). Metformin is currently being evaluated for disease control in AAA in several RCTs (94). Studies evaluating the benefits of metformin in NS-TAD are required.

#### 3.12.3 Should lipid lowering vs. placebo be used for NS-TAD?

##### 3.12.3.1 Recommendation

We cannot make a recommendation for lipid lowering in NS-TAD (Very low certainty of evidence).

##### 3.12.3.2 Evidence summary

Lipid-lowering drugs improve survival in people with atherosclerotic cardiovascular disease. There is no evidence specific to NS-TAD. A small (*n* = 36) experimental-medicine trial demonstrated 20-mg of atorvastatin significantly reduced low-density lipoprotein (LDL)-cholesterol levels (−47%) and induced regression of thoracic and abdominal aortic plaques (−15%) measured by MRI at 2 years in people with hypercholesterolaemia (95, 96). Applicability of this evidence is limited to degenerative NS-TAD, a disease of older people, that is not the target of cascade screening. The evidence is also of low certainty given the small sample size and the low number of clinical events.

##### 3.12.3.3 Justification

Lipid control could be considered in this patient population for the management of cardiovascular disease risk. From the patients' perspective, although the effect in slowing disease progression is likely to be small, this was considered a valuable outcome. Nonetheless, potential side effects were considered debilitating as well. In conclusion, given the level of evidence, and the indirectness of the evidence, we cannot recommend the routine use of statins in the NS-TAD population.

##### 3.12.3.4 Implementation considerations

Statins are generally well tolerated by patients, but they may have some side effects such as muscle pain, liver damage or diabetes. The cost of statins may vary depending on the type, dose and availability of the drug in different countries and regions.

##### 3.12.3.5 Research priorities

More research is needed to determine the biological plausibility and effectiveness of lipid lowering in TAD.

## 4 Discussion

To our knowledge, this document represents the first clinical practice guideline for thoracic aortic disease developed through a direct partnership with patients, relatives, and the public, for whom the recommendations are intended. This multi-stakeholder consensus exercise utilised the rigorous GRADE methodology to address key uncertainties in the screening, surveillance, and prevention of NS-TAD, producing seven recommendations and identifying five areas requiring prioritised research. The process also showed a significant gap between the clinical need for evidence and the available high-quality research, with most recommendations being conditional and based on low or very low certainty evidence.

A key finding of this study was the strong recommendation for non-selective imaging screening in first-degree relatives of patients with non-syndromic TAD. This is founded on evidence synthesised from observational studies indicating a diagnostic yield for aortic aneurysm of over 25% in this group, a rate substantially higher than the incidental diagnosis rate of approximately 2% in the general population (27). This recommendation aligns with existing international guidelines. All current guidelines encourage screening although with different implementation considerations (6, 11, 97). The 2022 ACC/AHA Guideline for the Diagnosis and Management of Aortic Disease gives a Class 1 (strong) recommendation for imaging of FDRs of patients with aortic aneurysm or dissection to identify asymptomatic disease (4). Similarly, the 2024 ESC Guidelines for the management of aortic diseases provide a Class I recommendation for screening all FDRs of patients with risk factors for heritable forms of the disease (6). Our work reinforces this consensus, but uniquely adds the perspective that patients and relatives might perceive significant value in the reassurance of a negative test, an outcome that is often overlooked in purely clinical evaluations.

In pharmacotherapy, our conditional recommendations for the use of Angiotensin Receptor Blockers (ARBs) and beta-blockers are consistent with established guidance for patients with Marfan syndrome,. Both the ACC/AHA and ESC guidelines recommend medical therapy with either beta-blockers or ARBs to reduce the rate of aortic root enlargement in patients with Marfan syndrome (Class 1 and Class IIa recommendations, respectively). Our findings support this, but also highlight the low certainty of the evidence and the considerable heterogeneity in trial populations, such as the strong genotype-phenotype correlation in drug response seen in *FBN1* mutations (98). This emphasises that a non-individualised approach is suboptimal, further highlighting the need for a precision medicine strategy in TAD management.

The most novel aspect of this work is its methodology, which embedded patients and the public as equal partners throughout the research process. This approach carries intrinsic value beyond the resulting recommendations. By involving lay members from the outset, the research questions selected for evaluation were those that mattered most to the affected community, as evidenced by the ranking of PICOs which prioritised screening and prevention over other topics (15). This patient-centred prioritisation ensures that research efforts are directed toward areas of greatest potential impact on quality of life and patient-important outcomes. Moreover, direct involvement fosters a deeper understanding of the trade-offs that patients are willing to accept; for instance, the panel judged the potential for reassurance from screening to outweigh the psychological stress of testing, a nuance that may be missed in clinician-led processes. Such co-production is critical for developing decision support tools that are trusted and utilised, ultimately facilitating shared decision-making and improving the equity and relevance of care.

This study is not without limitations. The primary limitation is the availability of high-quality evidence to answer many of the clinical questions, forcing reliance on observational data or indirect evidence from different patient populations (e.g., abdominal aortic aneurysm for decision support tools). This resulted in low or very low certainty of evidence for most outcomes and led to several conditional recommendations or an inability to make a recommendation at all, particularly regarding the management of pregnancy and the specifics of genetic counselling. We also acknowledge that while the consensus panel was multidisciplinary, it was UK-centric, and implementation issues may vary in other healthcare systems.

## 5 Conclusion

Despite several limitations due to the poor quality of the available evidence and the broad nature of the underlying research question, our consensus exercise highlighted the lack of clear, unanimous clinical pathways to assist clinicians in managing patients affected by TAD. Policy development should expand the current recommendations with the integration of future scientific development that will likely allow for a precision-medicine approach and bring them closer to the public to allow shared decision making.

Concurrently, further research is needed both to help develop findings with an immediate clinical value (interpretation of genetic tests, functional imaging, drug therapy), and to explore implementation issues of the ones currently available but not unanimously adopted.
