# Supplementary material for: Co-produced evidence-based recommendations for cascade screening and secondary prevention in the relatives of people diagnosed with non-syndromic thoracic aortic disease
Source: Front Cardiovasc Med. 2026 Mar 31;13:1724843. doi: 10.3389/fcvm.2026.1724843 (PMC13076464; doi:10.3389/fcvm.2026.1724843)
Supplement: Supplementary file 1 [file Datasheet1.docx]

**Co-produced evidence-based recommendations for cascade screening, and secondary prevention in the relatives of people diagnosed with non-syndromic thoracic aortic disease**

**Supplementary Materials**

Contents

[Supplementary Figure 1 - Risk of Bias plot for the individual trials 5](#_Toc224733916)

[Supplementary Figure 2 - Risk of Bias summary for individual trials included in the consensus exercise 7](#_Toc224733917)

[Supplementary Figure 3 – Forest Plot describing the effect of different screening modalities on diagnosis in non-syndromic thoracic aortic diseases 8](#_Toc224733918)

[Supplementary Figure 4 - Forest Plot describing the role of different screening modalities on uptake in non-syndromic thoracic aortic diseases 9](#_Toc224733919)

[Supplementary Figure 5 - Forest Plot describing the role of different genetic screening modalities on diagnosis in non-syndromic thoracic aortic diseases 10](#_Toc224733920)

[Supplementary Figure 6 - Forest Plot describing the role of different genetic screening modalities on uptake in non-syndromic thoracic aortic diseases 11](#_Toc224733921)

[Supplementary Figure 7 - Forest Plot describing the effect of ARB on mortality in TAD 12](#_Toc224733922)

[Supplementary Figure 8 - Forest Plot describing the effect of ARB on incidence of acute aortic syndromes in TAD 14](#_Toc224733923)

[Supplementary Figure 9 - Forest Plot describing the effect of ARB on need for surgery in TAD 15](#_Toc224733924)

[Supplementary Figure 10- Forest Plot describing the effect of ARB on rate of change in TAD 16](#_Toc224733925)

[Supplementary Figure 11 - Forest Plot describing the effect of B-blocker on mortality in TAD 17](#_Toc224733926)

[Supplementary Figure 12 - Forest Plot describing the effect of B-blocker on incidence of acute aortic syndromes in TAD 18](#_Toc224733927)

[Supplementary Figure 13 - Forest Plot describing the effect of B-blocker on disease progression in TAD 19](#_Toc224733928)

[Supplementary Table 1 – Ranking of PICOs for the consensus exercise 20](#_Toc224733929)

[Supplementary Table 2 - Definitions and Scope of the Guideline Recommendations 21](#_Toc224733930)

[Supplementary Table 3 – PRISMA 2020 flow diagram for the systematic review 22](#_Toc224733931)

[Supplementary Table 4 - Risk of Bias summary for observational studies included in the consensus exercise 23](#_Toc224733932)

[Appendix 25](#_Toc224733933)

[Agree 2016 Reporting Checklist 26](#_Toc224733934)

[Search Strategy for the systematic reviews 30](#_Toc224733935)

# Supplementary Figure 1 - Risk of Bias plot for the individual trials


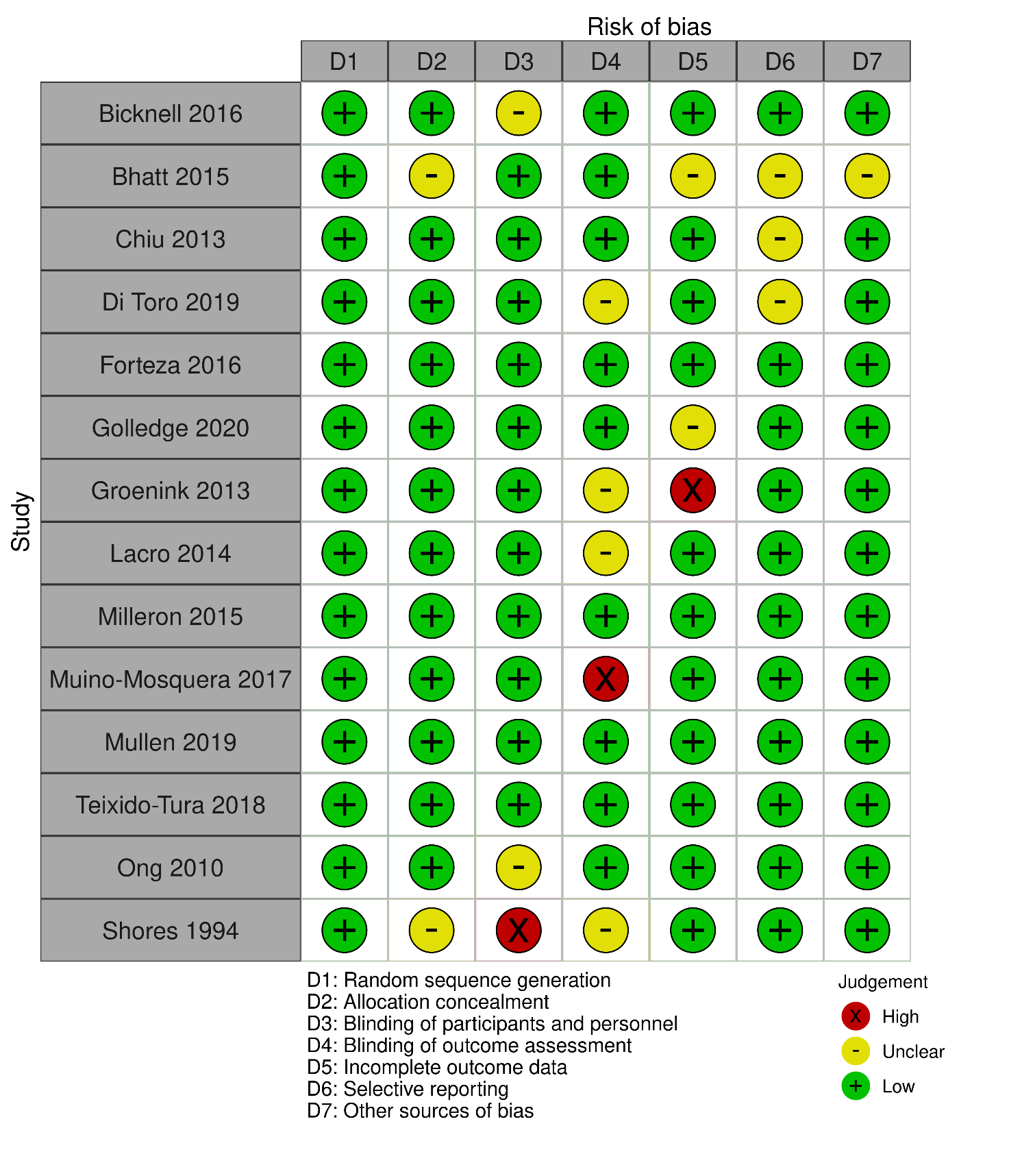


Plot reporting the risk of bias assessment for the individual trials included in the evidence synthesis exercise, according to Cochrane’s risk of bias tool, version 1 (Higgins JP, Altman DG, Gøtzsche PC, Jüni P, Moher D, Oxman AD, Savović J, Schulz KF, Weeks L, Sterne JA. The Cochrane Collaboration’s tool for assessing risk of bias in randomised trials. Bmj. 2011 Oct 18;343.).

Plot produced via the robvis tool (McGuinness, LA, Higgins, JPT. Risk-of-bias VISualization (robvis): An R package and Shiny web app for visualizing risk-of-bias assessments. Res Syn Meth. 2020; 1- 7. https://doi.org/10.1002/jrsm.1411)

# Supplementary Figure 2 - Risk of Bias summary for individual trials included in the consensus exercise


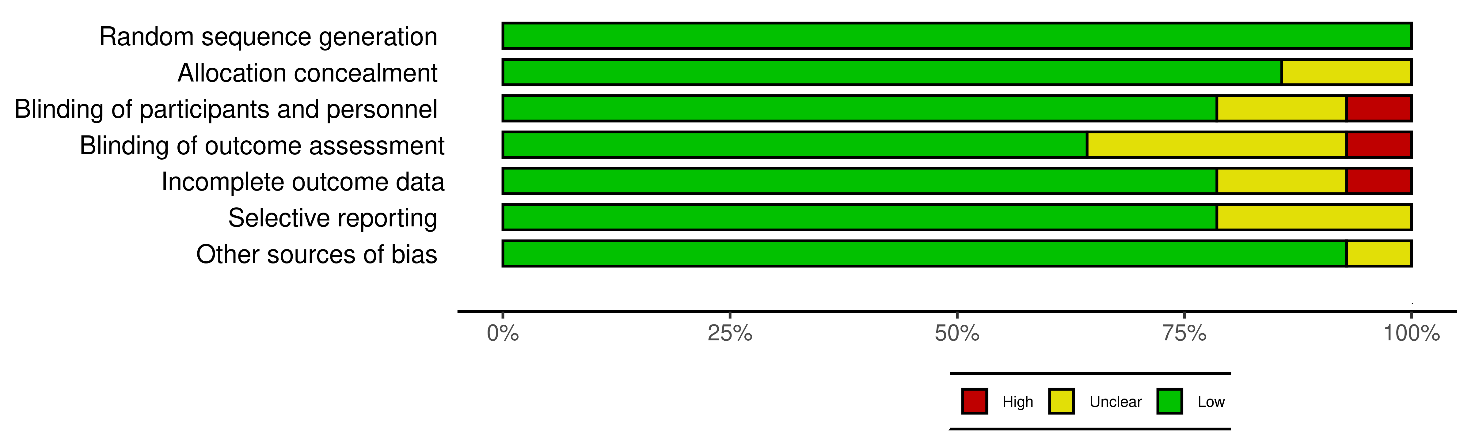


Plot summarising the overall risk of bias for the trials included in the evidence synthesis exercise, for the domains considered in Cochrane’s risk of bias tool, version 1 (Higgins JP, Altman DG, Gøtzsche PC, Jüni P, Moher D, Oxman AD, Savović J, Schulz KF, Weeks L, Sterne JA. The Cochrane Collaboration’s tool for assessing risk of bias in randomised trials. Bmj. 2011 Oct 18;343.).

Plot produced via the robvis tool (McGuinness, LA, Higgins, JPT. Risk-of-bias VISualization (robvis): An R package and Shiny web app for visualizing risk-of-bias assessments. Res Syn Meth. 2020; 1- 7. https://doi.org/10.1002/jrsm.1411)

# Supplementary Figure 3 – Forest Plot describing the effect of different screening modalities on diagnosis in non-syndromic thoracic aortic diseases


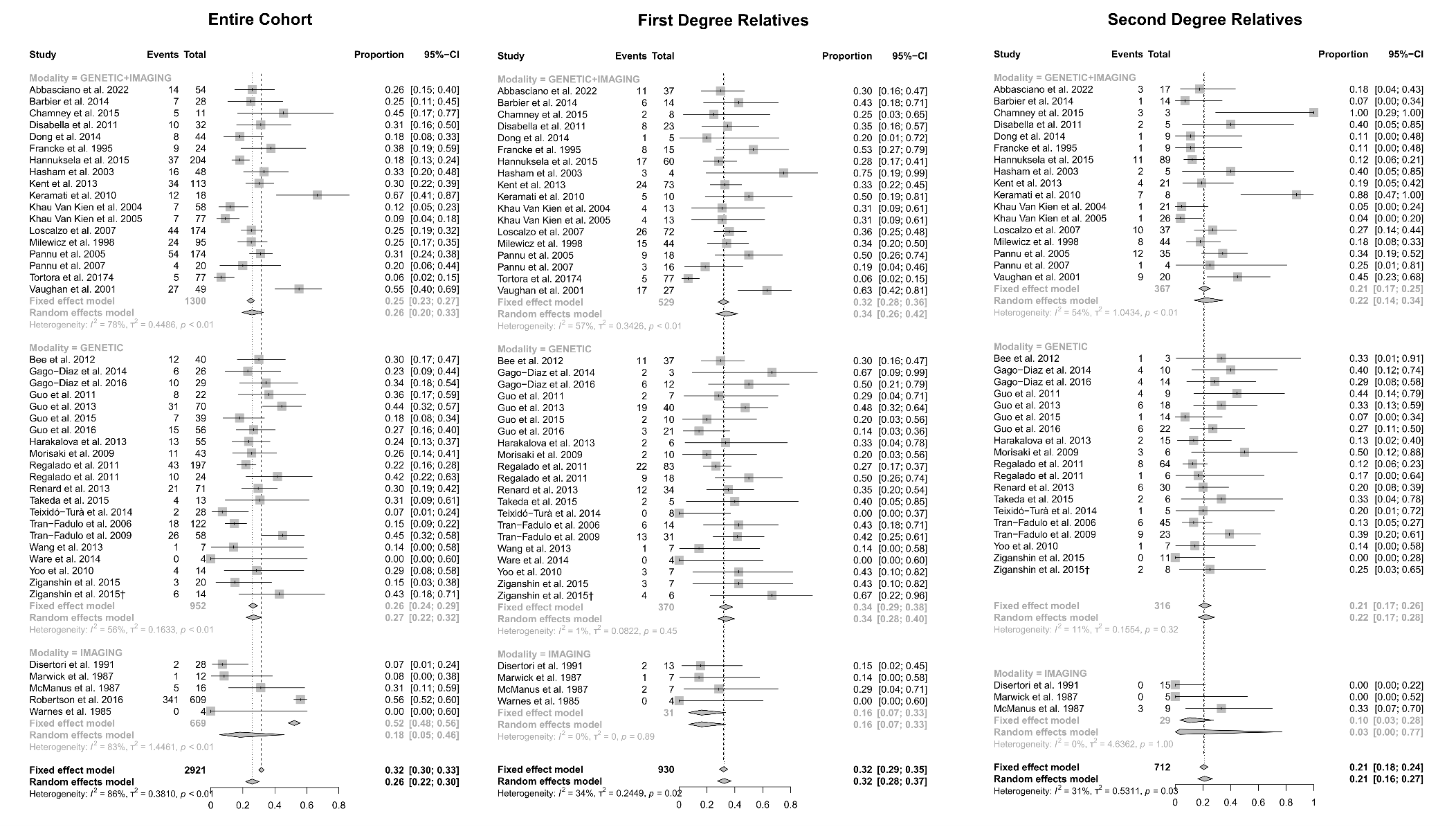


The forest plots report the results from the proportional meta-analysis of observational studies conducted in families in which one or more relatives were diagnosed with a non-syndromic thoracic aortic disease. The different studies (grouped based on the testing modality) are compared in terms of number of diagnoses, and the results are pooled both in a fixed effect and random effect model. *CI – Confidence Interval*

# Supplementary Figure 4 - Forest Plot describing the role of different screening modalities on uptake in non-syndromic thoracic aortic diseases


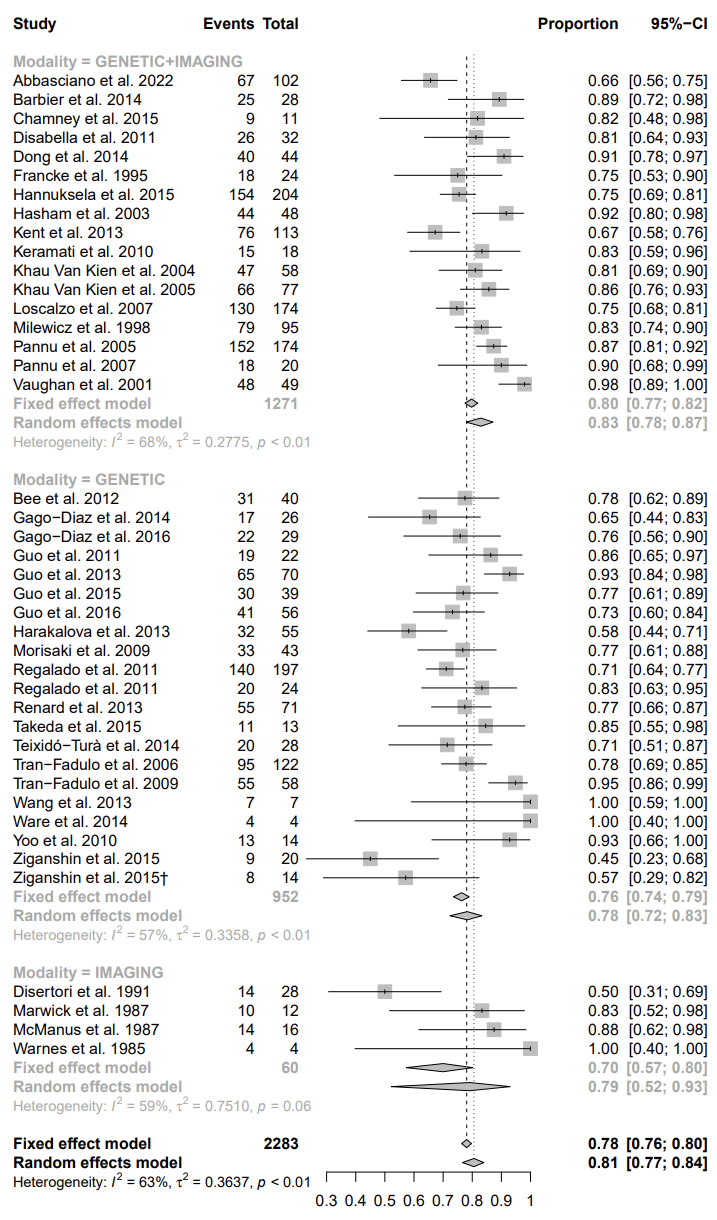


The forest plots report the results from the proportional meta-analysis of observational studies conducted in families in which one or more relatives were diagnosed with a non-syndromic thoracic aortic disease. The different studies (grouped based on the testing modality) are compared in terms of uptake of the screening offer, and the results are pooled both in a fixed effect and random effect model. *CI – Confidence Interval*

# Supplementary Figure 5 - Forest Plot describing the role of different genetic screening modalities on diagnosis in non-syndromic thoracic aortic diseases


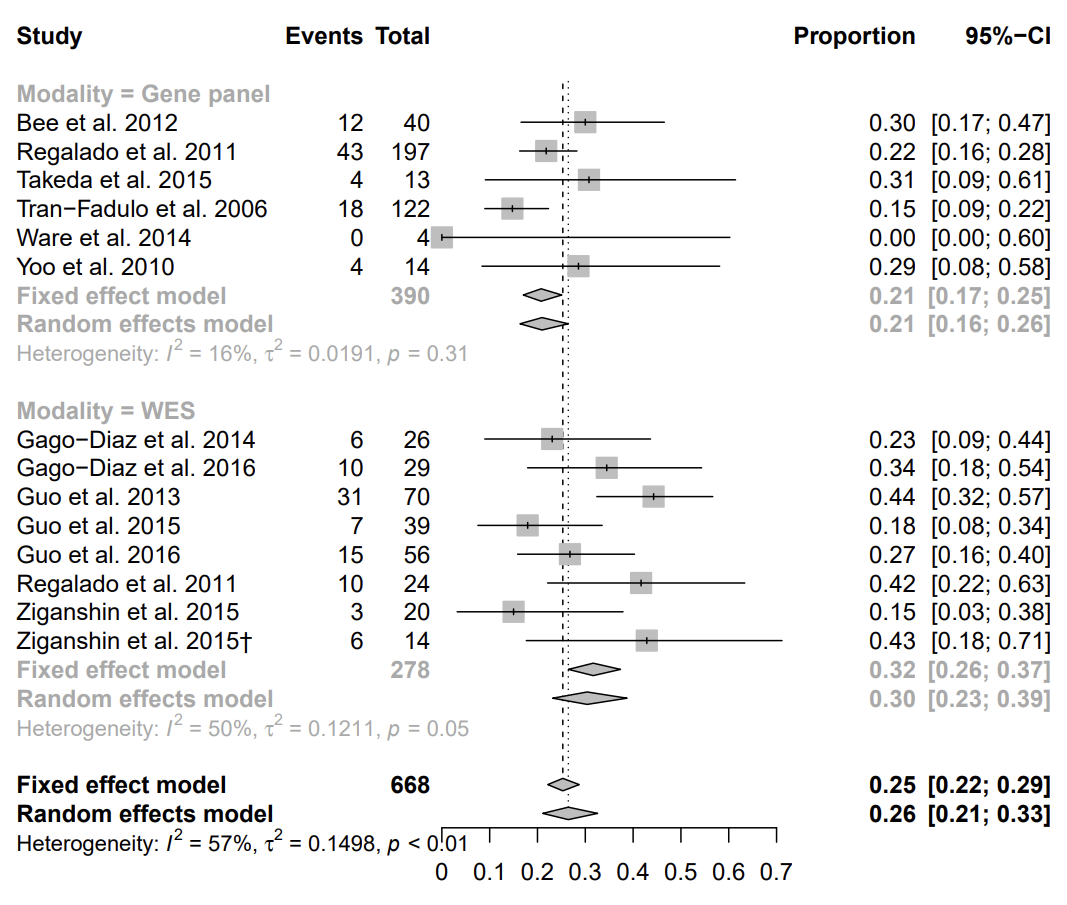


The forest plots report the results from the proportional meta-analysis of observational studies conducted in families in which one or more relatives were diagnosed with a non-syndromic thoracic aortic disease. The different studies (grouped based on the testing modality, gene panel or whole exome sequencing) are compared in terms of number of diagnoses, and the results are pooled both in a fixed effect and random effect model. *CI – Confidence Interval; WES – whole exome sequencing.*

# Supplementary Figure 6 - Forest Plot describing the role of different genetic screening modalities on uptake in non-syndromic thoracic aortic diseases


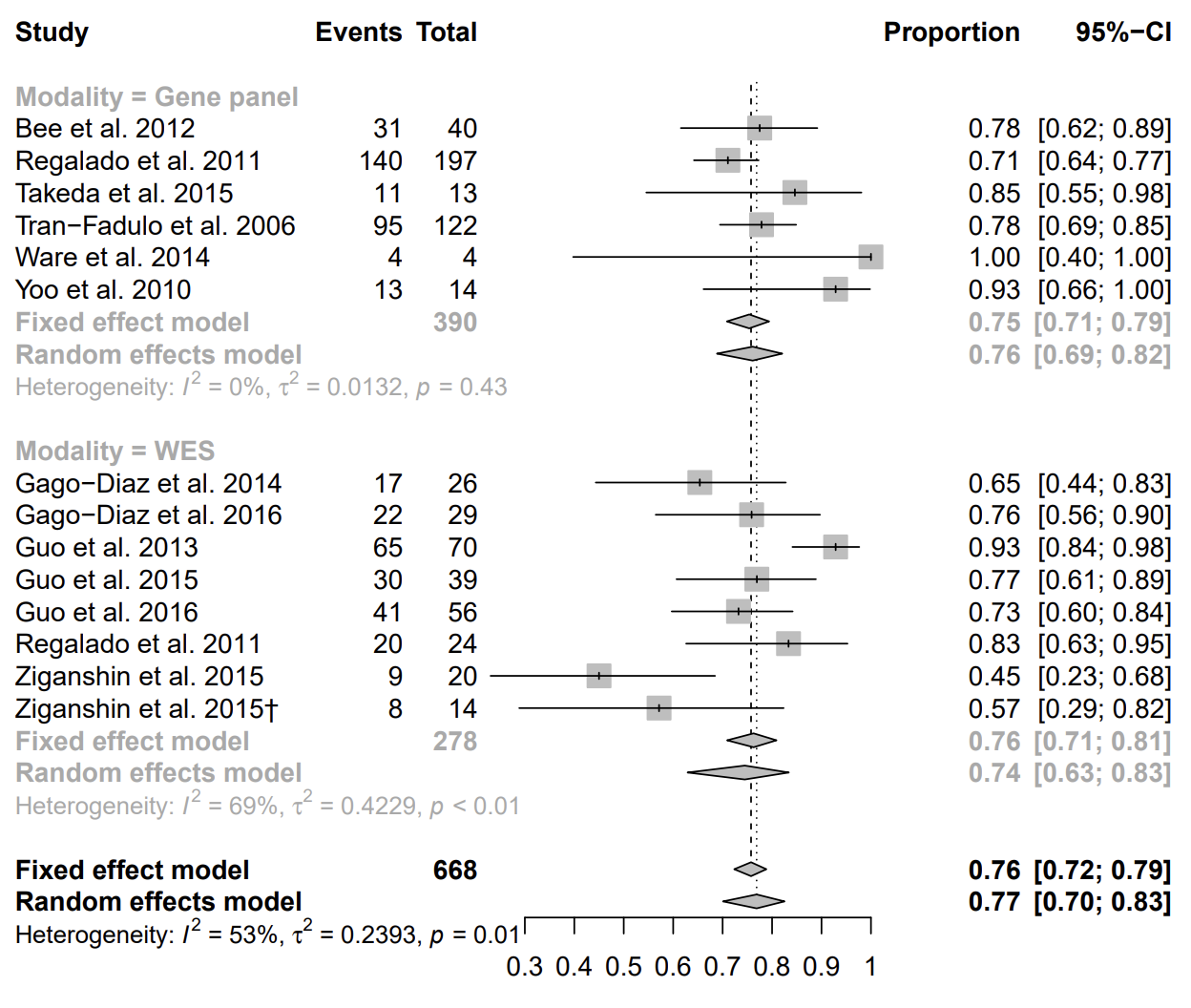


The forest plots report the results from the proportional meta-analysis of observational studies conducted in families in which one or more relatives were diagnosed with a non-syndromic thoracic aortic disease. The different studies (grouped based on the testing modality, gene panel or whole exome sequencing) are compared in terms of uptake of the screening offer, and the results are pooled both in a fixed effect and random effect model. *CI – Confidence Interval; WES – whole exome sequencing.*

# Supplementary Figure 7 - Forest Plot describing the effect of ARB on mortality in TAD

**
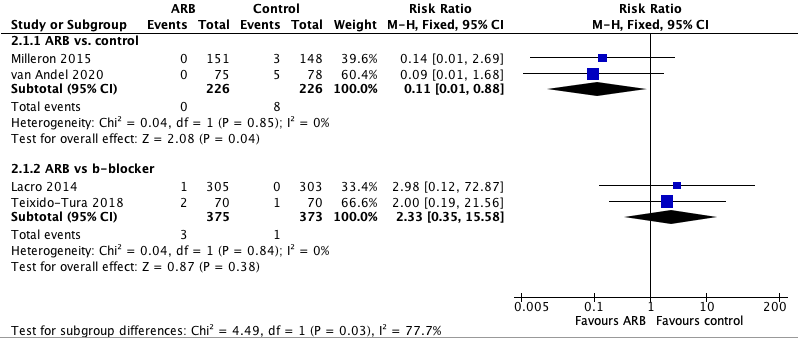
**

The forest plots report the results from the meta-analysis of randomised controlled trials conducted in patients affected by thoracic aortic disease. The different studies (grouped based on the comparison, control or beta-blocker) are compared in terms of mortality, and the results are pooled a fixed effect model. *ARB – Angiotensin Receptor Blocker; CI – Confidence Interval; TAD – Thoracic Aortic Disease.*

# Supplementary Figure 8 - Forest Plot describing the effect of ARB on incidence of acute aortic syndromes in TAD

**
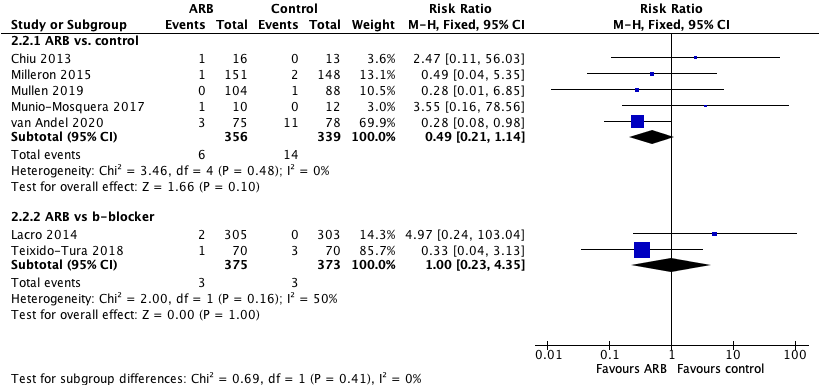
**

The forest plots report the results from the meta-analysis of randomised controlled trials conducted in patients affected by thoracic aortic disease. The different studies (grouped based on the comparison, control or beta-blocker) are compared in terms of incidence of acute aortic syndrome, and the results are pooled a fixed effect model. *ARB – Angiotensin Receptor Blocker; CI – Confidence Interval; TAD – Thoracic Aortic Disease.*

# Supplementary Figure 9 - Forest Plot describing the effect of ARB on need for surgery in TAD

**
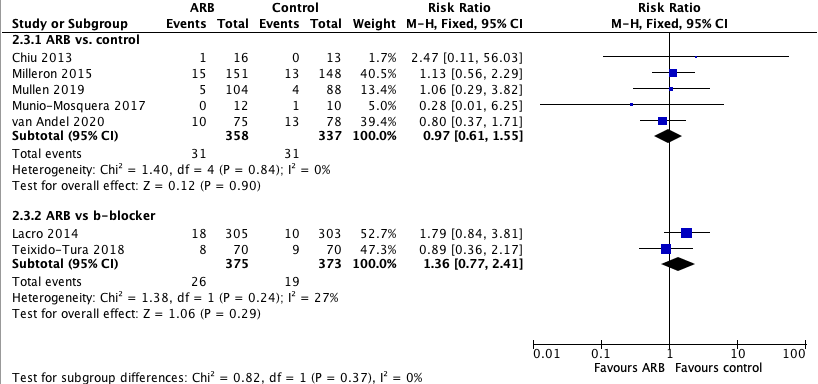
**

The forest plots report the results from the meta-analysis of randomised controlled trials conducted in patients affected by thoracic aortic disease. The different studies (grouped based on the comparison, control or beta-blocker) are compared in terms of incidence of acute aortic syndrome, and the results are pooled a fixed effect model. *ARB – Angiotensin Receptor Blocker; CI – Confidence Interval; TAD – Thoracic Aortic Disease.*

# Supplementary Figure 10- Forest Plot describing the effect of ARB on rate of change in TAD

**
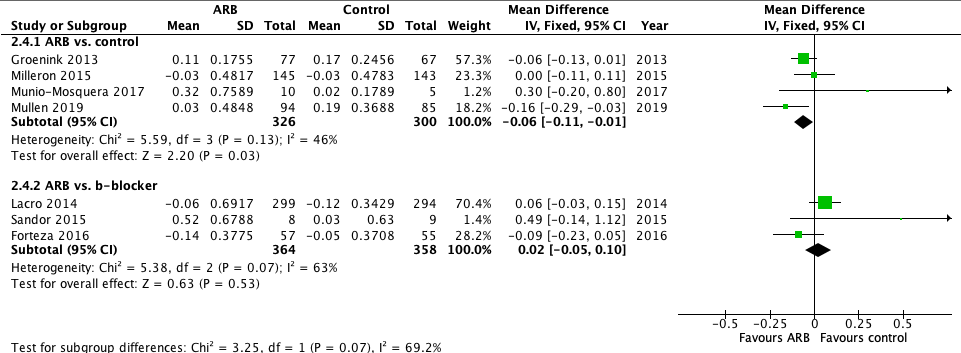
**

The forest plots report the results from the meta-analysis of randomised controlled trials conducted in patients affected by thoracic aortic disease. The different studies (grouped based on the comparison, control or beta-blocker) are compared in terms of rate of change (measured as body surface area-adjusted aortic root dimension Z score at the sinuses of Valsalva), and the results are pooled as mean difference in a fixed effect model. *ARB – Angiotensin Receptor Blocker; CI – Confidence Interval; TAD – Thoracic Aortic Disease.*

# Supplementary Figure 11 - Forest Plot describing the effect of B-blocker on mortality in TAD

**
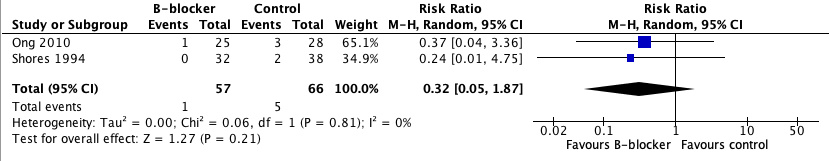
**

The forest plots report the results from the meta-analysis of randomised controlled trials conducted in patients affected by thoracic aortic disease. The different studies are compared in terms of rate of mortality, and the results are pooled as risk ratio in a random effect model. *CI – Confidence Interval; TAD – Thoracic Aortic Disease.*

# Supplementary Figure 12 - Forest Plot describing the effect of B-blocker on incidence of acute aortic syndromes in TAD

**
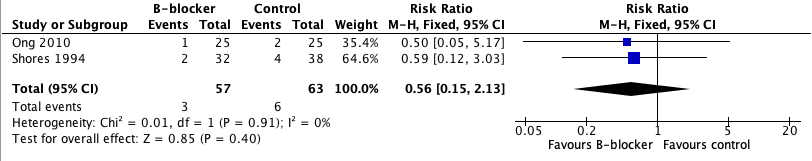
**

The forest plots report the results from the meta-analysis of randomised controlled trials conducted in patients affected by thoracic aortic disease. The different studies are compared in terms of rate of incidence of acute aortic syndromes, and the results are pooled as risk ratio in a random effect model. *CI – Confidence Interval; TAD – Thoracic Aortic Disease.*

# Supplementary Figure 13 - Forest Plot describing the effect of B-blocker on disease progression in TAD

**
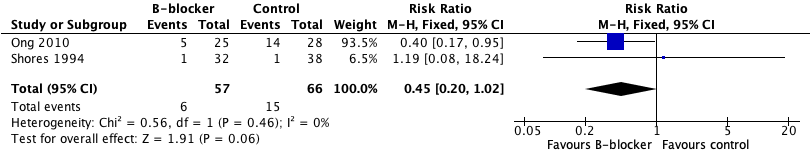
**

The forest plots report the results from the meta-analysis of randomised controlled trials in patients affected by thoracic aortic disease. The different studies are compared in terms of rate of disease progression (defined as composite of cardiac or arterial events; or aortic root>6cm), and the results are pooled as risk ratio in a random effect model. *CI – Confidence Interval; TAD – Thoracic Aortic Disease.*

# Supplementary Table 1 – Ranking of PICOs for the consensus exercise


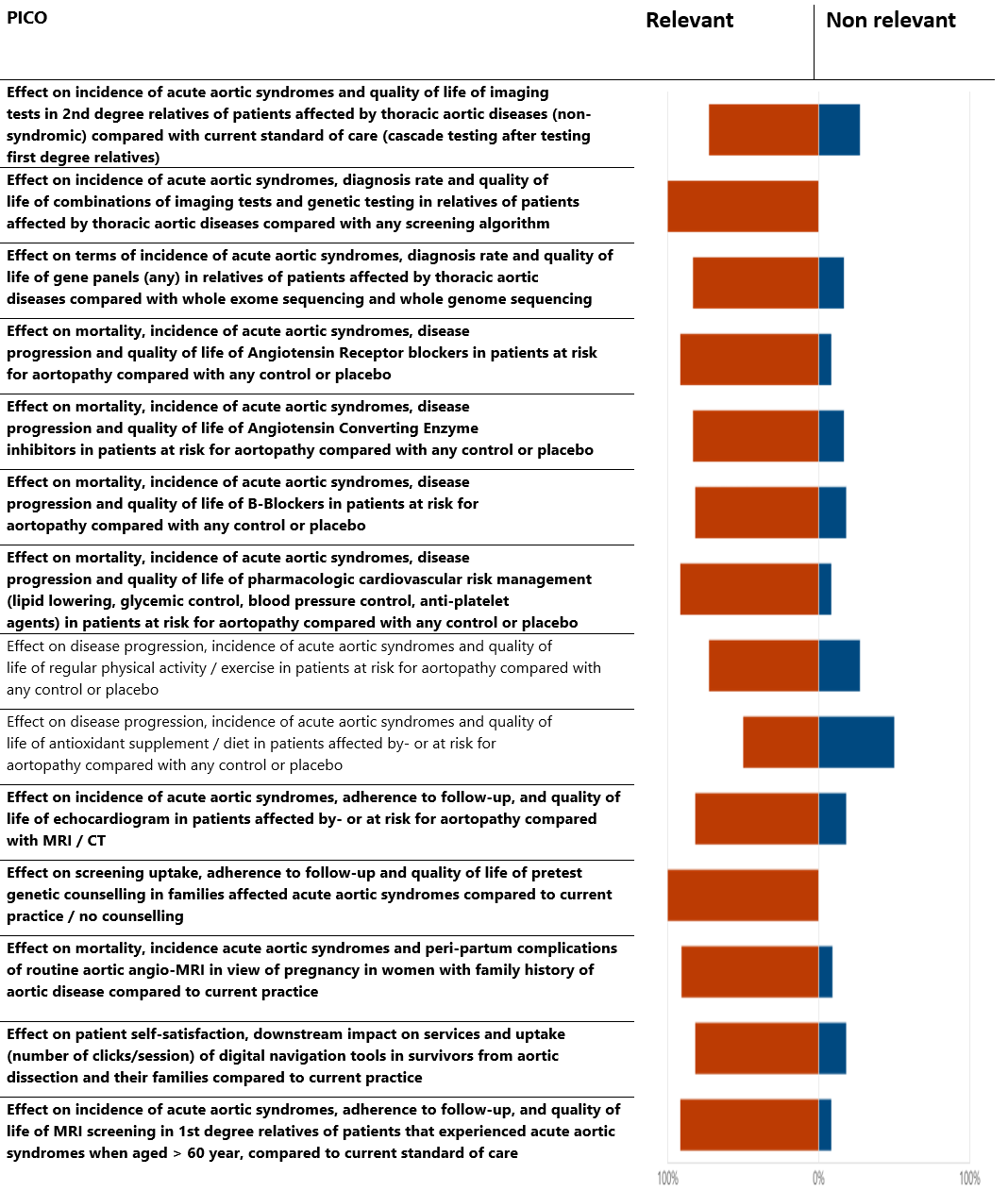


The table reports the last stage of the process that led to the prioritisation of the PICOs that made the final list of clinical questions that were explored by the consensus exercise.

# Supplementary Table 2 - Definitions and Scope of the Guideline Recommendations

| Term / Category | Definition & Inclusion Criteria | Exclusion / Out of Scope |
| --- | --- | --- |
| **NS-TAD (General)** | Thoracic Aortic Disease (aneurysm or dissection) where the disease phenotype is restricted to the aorta | Syndromic TAD (e.g., Marfan, Loeys-Dietz, Ehlers-Danlos) with extra-aortic manifestations |
| **Heritable NS-TAD** | NS-TAD in a proband with a previous documented case of TAD in the family |  |
| **Sporadic NS-TAD** | NS-TAD in a proband with no known family history of TAD |  |
| **Clinical Presentation** | Includes both patients with asymptomatic aortic aneurysm and survivors of Acute Aortic Syndromes (Dissection) |  |
| **Anatomical Scope** | Thoracic Aorta including: Aortic Root, Ascending Aorta, Aortic Arch, and Descending Thoracic Aorta | Abdominal Aortic Aneurysm (unless concurrent with thoracic disease). |
| **Bicuspid Aortic Valve** | Included when associated with thoracic aortic aneurysm/dilation |  |
| **Age Boundaries** | Adults >18 years |  |

# Supplementary Table 3 – PRISMA 2020 flow diagram for the systematic review

**Identification of studies via databases and registers**

Records removed *before screening*:

Duplicate records removed (n = 280)

7115 records identified from databases and registers

**Identification**

Records excluded

(n = 3390)

Records screened

(n = 6835)

Reports excluded:

Ineligible population (n = 13)

Ineligible intervention (n = 7)

Reports sought for retrieval

(n = 41)

**Screening**

Reports included from Mariscalco 2018:

n = 53

74 studies included in the systematic review

**Included**

67 studies included in the meta-analysis

The flow diagram describes the identification, screening and inclusion process for the studies selected for the consensus exercise.

# Supplementary Table 4 - Risk of Bias summary for observational studies included in the consensus exercise

| **Study (Author/Year)** | **Newcastle-Ottawa Scale** | | |
| --- | --- | --- | --- |
|  | **Selection** | **Comparability** | **Outcome** |
| Abbasciano et al. 2022 | ☆☆☆ | ☆☆☆ | ☆☆ |
| Barbier et al. 2014 | ☆☆ | ☆☆ | ☆ |
| Bee et al. 2012 | ☆ | ☆☆ | ☆☆☆ |
| Chamney et al. 2015 | ☆ | ☆ | ☆☆ |
| Disabella et al. 2011 | ☆☆☆ | ☆☆ | ☆☆☆ |
| Disertori et al. 1991 | - | - | ☆ |
| Dong et al. 2014 | ☆☆☆ | ☆☆ | ☆☆☆ |
| Francke et al. 1995 | ☆☆ | ☆ | ☆☆☆ |
| Gago-Diaz et al. 2014 | ☆☆☆ | ☆☆ | ☆☆☆ |
| Gago-Diaz et al. 2016 | ☆☆ | ☆☆ | ☆☆ |
| Guo et al. 2001 | ☆ | ☆ | ☆☆☆ |
| Guo et al. 2007 | ☆☆ | ☆ | ☆☆☆ |
| Guo et al. 2009 | ☆☆☆ | ☆ | ☆☆☆ |
| Guo et al. 2011 | ☆☆☆ | ☆ | ☆☆☆ |
| Guo et al. 2013 | ☆☆☆ | ☆ | ☆☆☆ |
| Guo et al. 2015 | ☆☆☆ | ☆ | ☆☆☆ |
| Guo et al. 2016 | ☆☆☆ | ☆ | ☆☆ |
| Hannuksela et al. 2015 | ☆☆☆ | ☆ | ☆☆☆ |
| Hannuksela et al. 2016 | ☆☆☆ | ☆☆ | ☆☆ |
| Harakalova et al. 2013 | ☆☆☆ | ☆☆ | ☆☆☆ |
| Hasham et al. 2003 | ☆☆☆ | ☆☆ | ☆☆☆ |
| Kakko et al. 2003 | ☆☆☆ | ☆☆ | ☆☆ |
| Kent et al. 2013 | ☆☆ | ☆☆ | ☆☆☆ |
| Keramati et al. 2010 | ☆☆ | ☆ | ☆☆ |
| Khau Van Kien et al. 2004 | ☆☆☆ | ☆☆ | ☆☆☆ |
| Khau Van Kien et al. 2005 | ☆☆☆ | ☆☆ | ☆☆☆ |
| Kuang et al. 2016 | ☆☆☆ | ☆☆ | ☆ |
| Loscalzo et al. 2007 | ☆☆☆ | ☆ | ☆☆☆ |
| Marwick et al. 1987 | - | ☆ | ☆ |
| McManus et al. 1987 | - | ☆ | ☆☆ |
| Milewicz et al. 1998 | ☆☆ | ☆ | ☆☆ |
| Morisaki et al. 2009 | ☆☆ | ☆ | ☆ |
| Pannu et al. 2005 | ☆☆☆ | ☆☆ | ☆ |
| Pannu et al. 2007 | ☆☆☆ | ☆☆ | ☆☆☆ |
| Regalado et al. 2011 | ☆☆ | ☆☆ | ☆ |
| Regalado et al. 2011 | ☆☆ | ☆☆ | ☆☆ |
| Regalado et al. 2011 | ☆☆ | ☆☆ | ☆☆ |
| Renard et al. 2013 | ☆☆ | ☆☆ | ☆☆ |
| Robertson et al. 2016 | ☆☆☆ | ☆☆ | ☆☆☆ |
| Sherrah et al. 2016 | ☆☆☆ | ☆☆ | ☆☆☆ |
| Takeda et al. 2015 | ☆☆ | ☆☆ | ☆☆ |
| Teixidó-Turà et al. 2014 | ☆ | ☆ | ☆ |
| Tortora et al. 2017 | * | * | * |
| Tran-Fadulo et al. 2006 | ☆☆ | ☆ | ☆☆ |
| Tran-Fadulo et al. 2009 | ☆☆ | ☆☆ | ☆☆ |
| Vaughan et al. 2001 | ☆☆☆ | ☆☆ | ☆☆☆ |
| Wang et al. 2010 | ☆☆ | ☆ | ☆☆ |
| Wang et al. 2013 | ☆☆ | ☆☆ | ☆☆ |
| Ware et al. 2014 | ☆ | ☆ | ☆ |
| Warnes et al. 1985 | - | ☆ | ☆ |
| Weigang et al. 2007 | ☆☆☆ | ☆☆ | ☆☆ |
| Yoo et al. 2010 | ☆☆ | ☆☆ | ☆☆ |
| Zhu et al. 2006 | ☆☆☆ | ☆☆ | ☆☆ |
| Ziganshin et al. 2015 | ☆☆ | ☆ | ☆ |

The table reports the risk of bias judgements for the observational studies assessing screening modalities included in the evidence synthesis exercise, for the domains considered in the Newcaste-Ottawa scale (Wells G, Shea B, O'Connell D, Peterson j, Welch V, Losos M, et al. The Newcastle–Ottawa Scale (NOS) for Assessing the Quality of Non-Randomized Studies in Meta-Analysis, 2000).

For observational studies conducted before 2018, the ratings expressed in the review by Mariscalco et al. (Mariscalco G, Debiec R, Elefteriades JA, Samani NJ, Murphy GJ. JAHA. Systematic review of studies that have evaluated screening tests in relatives of patients affected by nonsyndromic thoracic aortic disease. 2018;7(15):e009302.) have been adopted.

# Appendix

## Agree 2016 Reporting Checklist

**
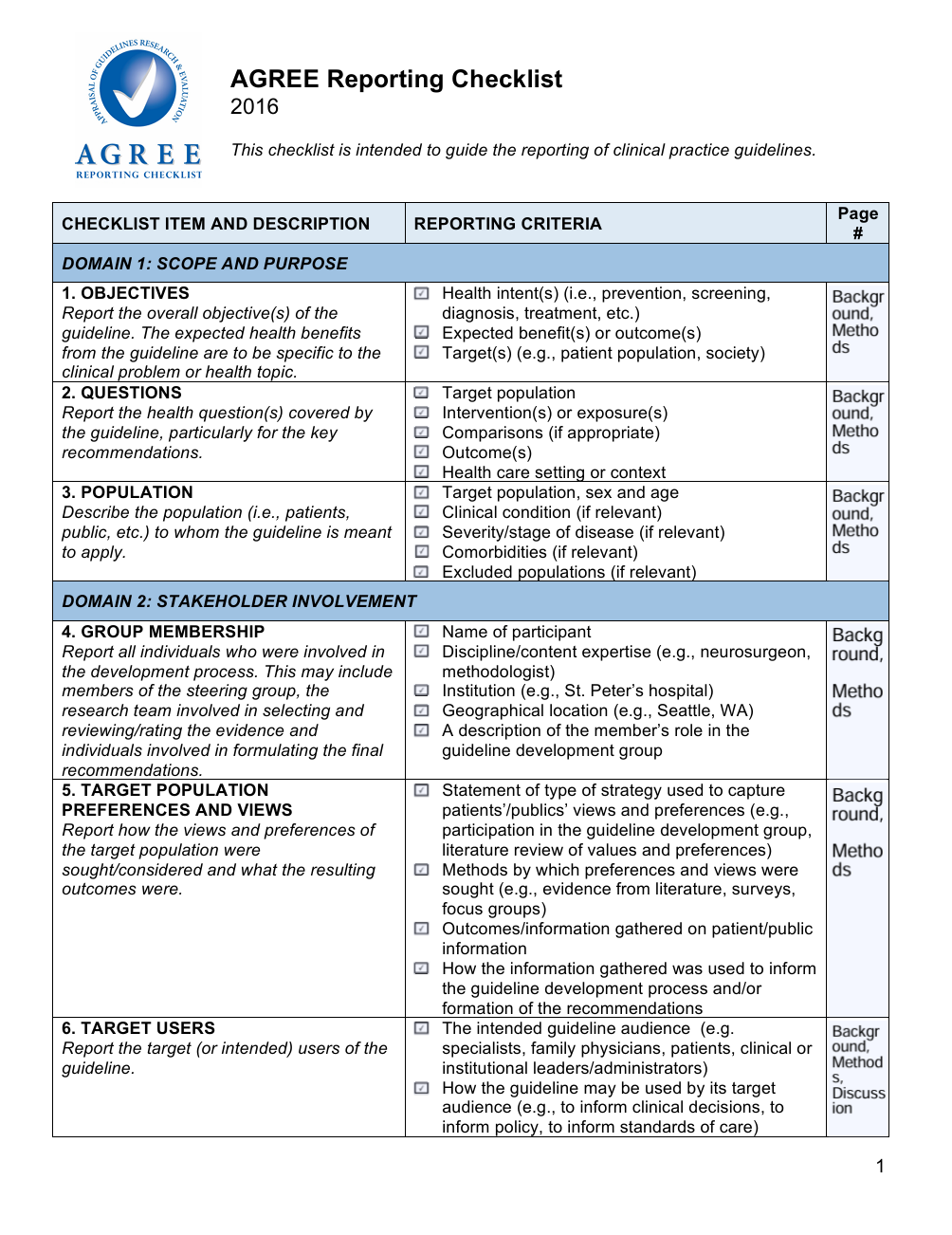
**

**
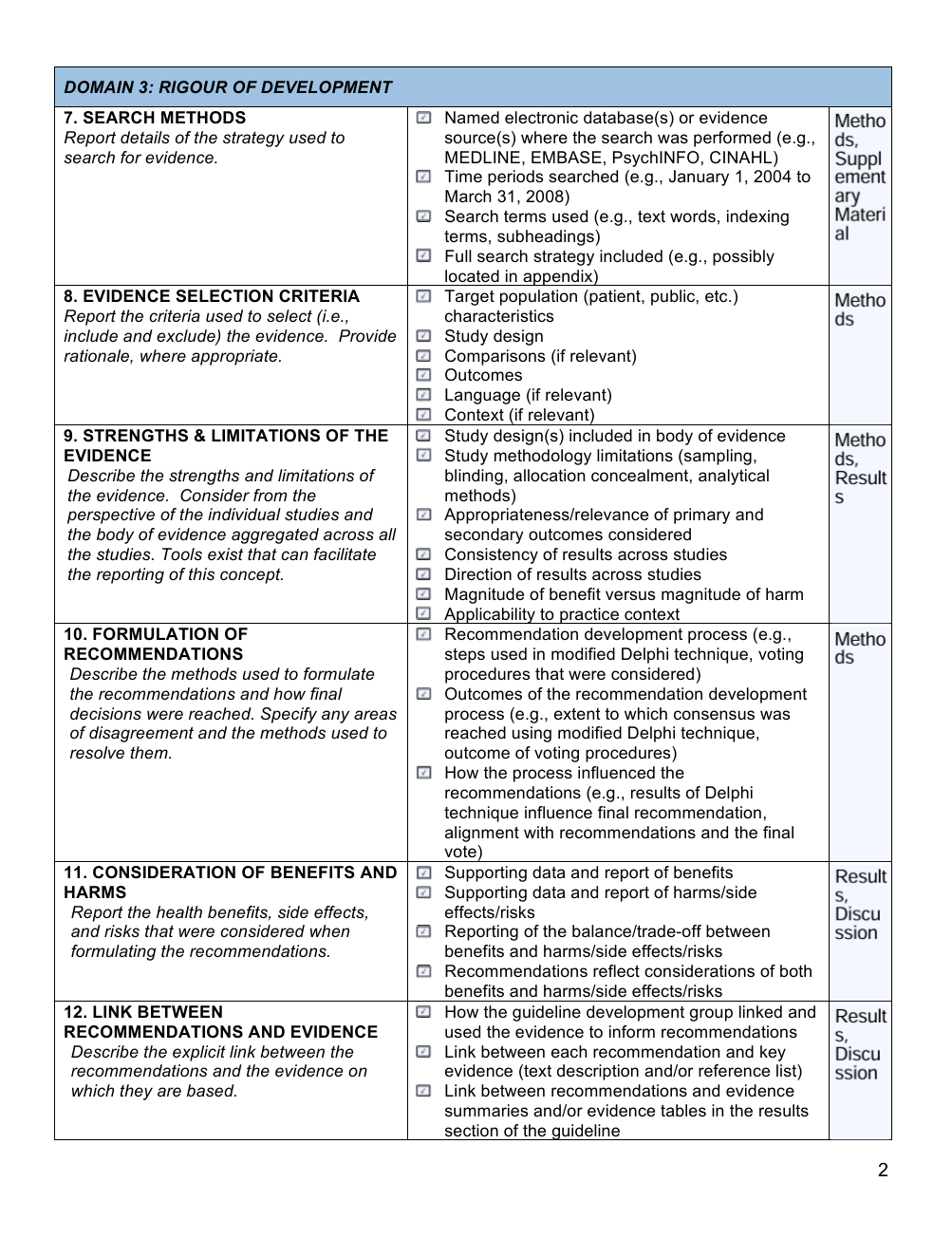
**

**
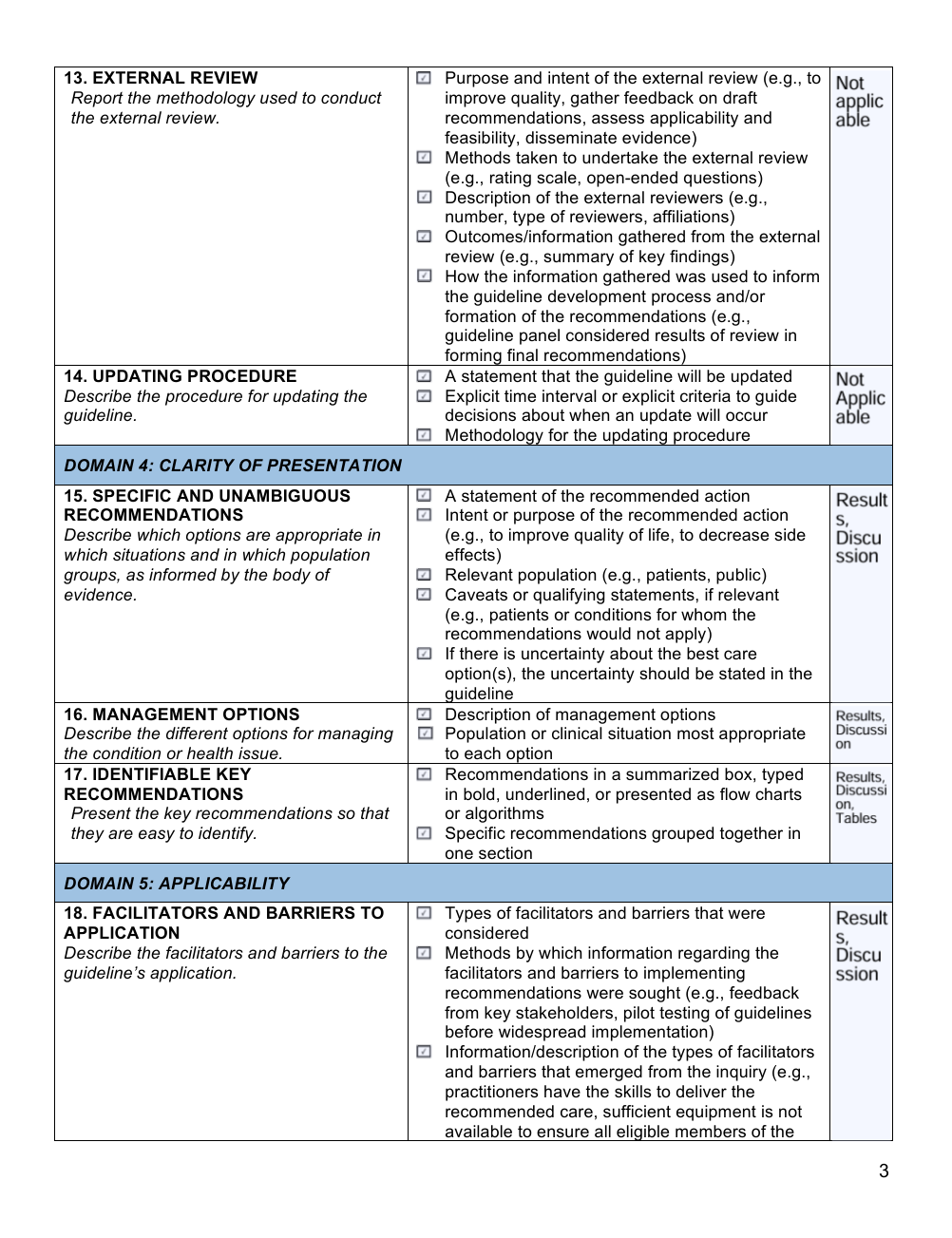
**

**
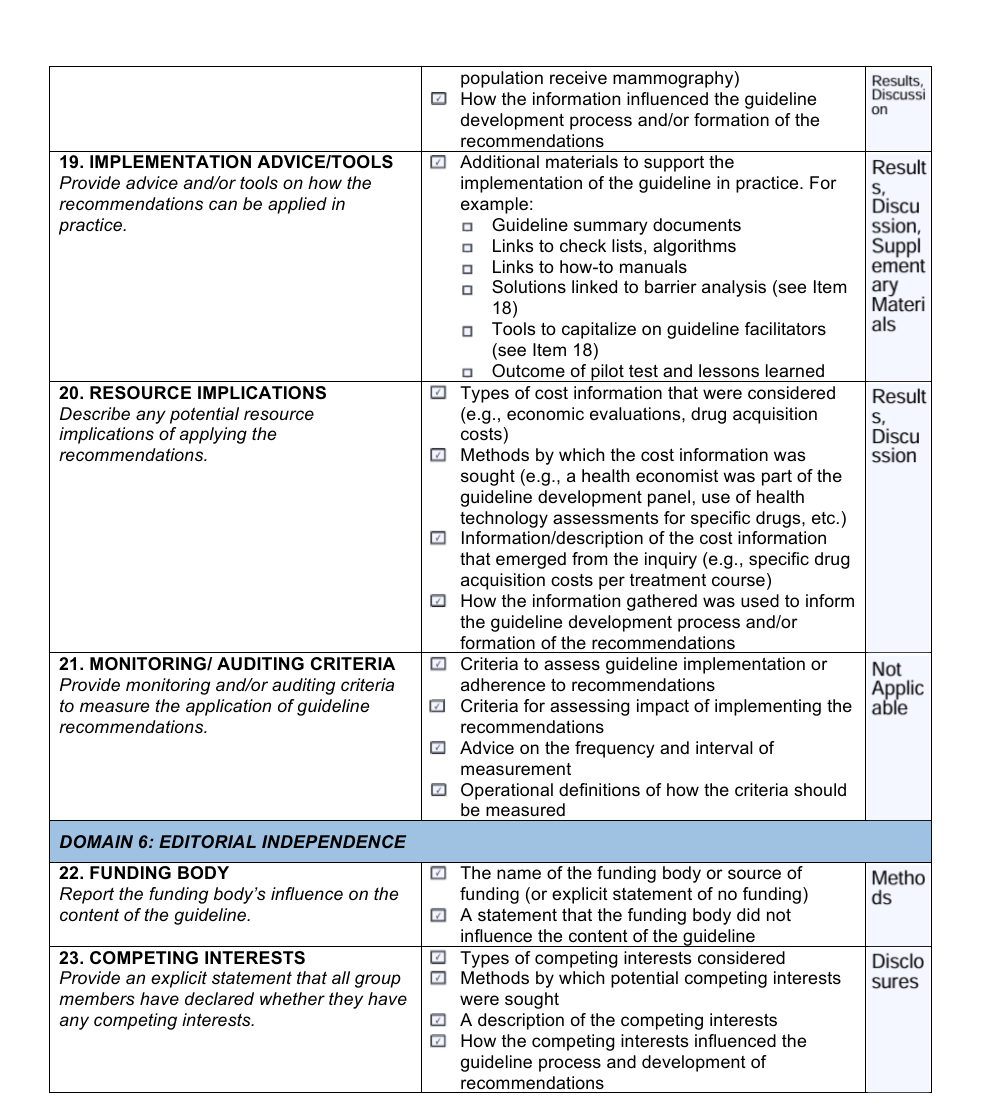
**

From: Brouwers MC, Kerkvliet K, Spithoff K, on behalf of the AGREE Next Steps Consortium. The AGREE Reporting Checklist: a tool to improve reporting of clinical practice guidelines. BMJ 2016;352:i1152. doi: 10.1136/bmj.i1152.

## Search Strategy for the systematic reviews

*Prevention*

MEDLINE through OVID (Run on 4th August 2021, adapted for other databases: Embase, CENTRAL)

1 (Aortic or Aorta).mp.

2 exp aorta/

3 1 or 2

4 exp Preventive Health Services/

5 (prevent* or prophyla*).mp.

6 4 or 5

7 3 and 6

8 limit 7 to (human and randomized controlled trial)

**2069** records retrieved

*Surveillance and Screening*

MEDLINE through OVID (Run on 6th August 2022, adapted for other databases: Embase, CENTRAL)

1 (Aortic or Aorta).mp.

2 exp aorta/

3 1 or 2

4 exp Secondary Care/

5 exp Diagnostic Screening Programs/

6 (follow-up or "follow up").ab.

7 surveillance.ti,ab.

8 screening.ti,ab.

9 4 or 5 or 6 or 7 or 8

10 3 and 9

11 limit 10 to (humans and randomized controlled trial and last 10 years)

**2149** records retrieved

*Guidelines and Consensus*

Searches performed on Medline (476 references) and Embase (2696 references) on the 21^st^ October 2020

1. aorta/ or thoracic aorta/ or ascending aorta surgery/ or ascending aorta/ or aorta.mp. or thoracic aorta aneurysm/

2. gene*.mp. or genetics/ or echocardiography.mp or computed tomography.mp or magnetic resonance.mp or US.mp or CT.mp or MRI.mp

3. (consensus or recommendation* or guideline* or expert).ti,ab.

4. (surveillance or management or therapy or test or indication or threshold).mp.

5. 1 and 2 and 3 and 4

**2897** records retrieved
